# Dexamethasone priming enhances stemness and immunomodulatory property of tissue-specific human mesenchymal stem cells

**DOI:** 10.1186/s12861-021-00246-4

**Published:** 2021-11-04

**Authors:** Sonali Rawat, Vatsla Dadhwal, Sujata Mohanty

**Affiliations:** 1 Stem Cell Facility, All India Institute of Medical Science, New Delhi, India; 2 Department of Obstetrics and Gynecology, All India Institute of Medical Science, New Delhi, India

**Keywords:** Mesenchymal stem cell, Immunomodulatory, Glucocorticoids, Dexamethasone, Autoimmune diseases, Anti-inflammatory, Allogenic

## Abstract

**Background:**

Human Mesenchymal Stem Cells (hMSCs) represent a promising cell source for cell-based therapy in autoimmune diseases and other degenerative disorders due to their immunosuppressive, anti-inflammatory and regenerative potentials. Belonging to a glucocorticoid family, Dexamethasone (Dex) is a powerful anti-inflammatory compound that is widely used as therapy in autoimmune disease conditions or allogeneic transplantation. However, minimal immunomodulatory effect of hMSCs may limit their therapeutic uses. Moreover, the effect of glucocorticoids on the immunomodulatory molecules or other regenerative properties of tissue-specific hMSCs remains unknown.

**Method:**

Herein, we evaluated the in vitro effect of Dex at various dose concentrations and time intervals, 1000 ng/ml, 2000 ng/ml, 3000 ng/ml and 24 h, 48 h respectively, on the basic characteristics and immunomodulatory properties of Bone marrow derived MSC (BM-MSCs), Adipose tissue derived MSCs (AD-MSCs), Dental Pulp derived MSC (DP-MSCs) and Umbilical cord derived MSCs (UC-MSCs).

**Results:**

The present study indicated that the concentration of Dex did not ramify the cellular morphology nor showed cytotoxicity as well as conserved the basic characteristics of tissue specific hMSCs including cell proliferation and surface marker profiling. However, quite interestingly it was observed that the stemness markers (Oct-4, Sox-2, Nanog and Klf-4) showed a significant upregulation in DP-MSCs and AD-MSCs followed by UC-MSCs and BM-MSCs. Additionally, immunomodulatory molecules, Prostaglandin E-2 (PGE-2), Indoleamine- 2,3-dioxygenase (IDO) and Human Leukocyte Antigen-G (HLA-G) were seen to be upregulated in a dose-dependent manner. Moreover, there was a differential response of tissue specific hMSCs after pre-conditioning with Dex during mixed lymphocyte reaction, wherein UC-MSCs and DP-MSCs showed enhanced immunosuppression as compared to AD-MSCs and BM-MSCs, thereby proving to be a better candidate for therapeutic applications in immune-related diseases.

**Conclusion:**

Dex preconditioning improved the hMSCs immunomodulatory property and may have reduced the challenge associated with minimal potency and strengthen their therapeutic efficacy.

**Graphical Abstract:**

Preconditioning of tissue specific hMSCs with dexamethasone biomanufacturers the enhanced potential hMSCs with better stemness and immunomodulatory properties for future therapeutics.
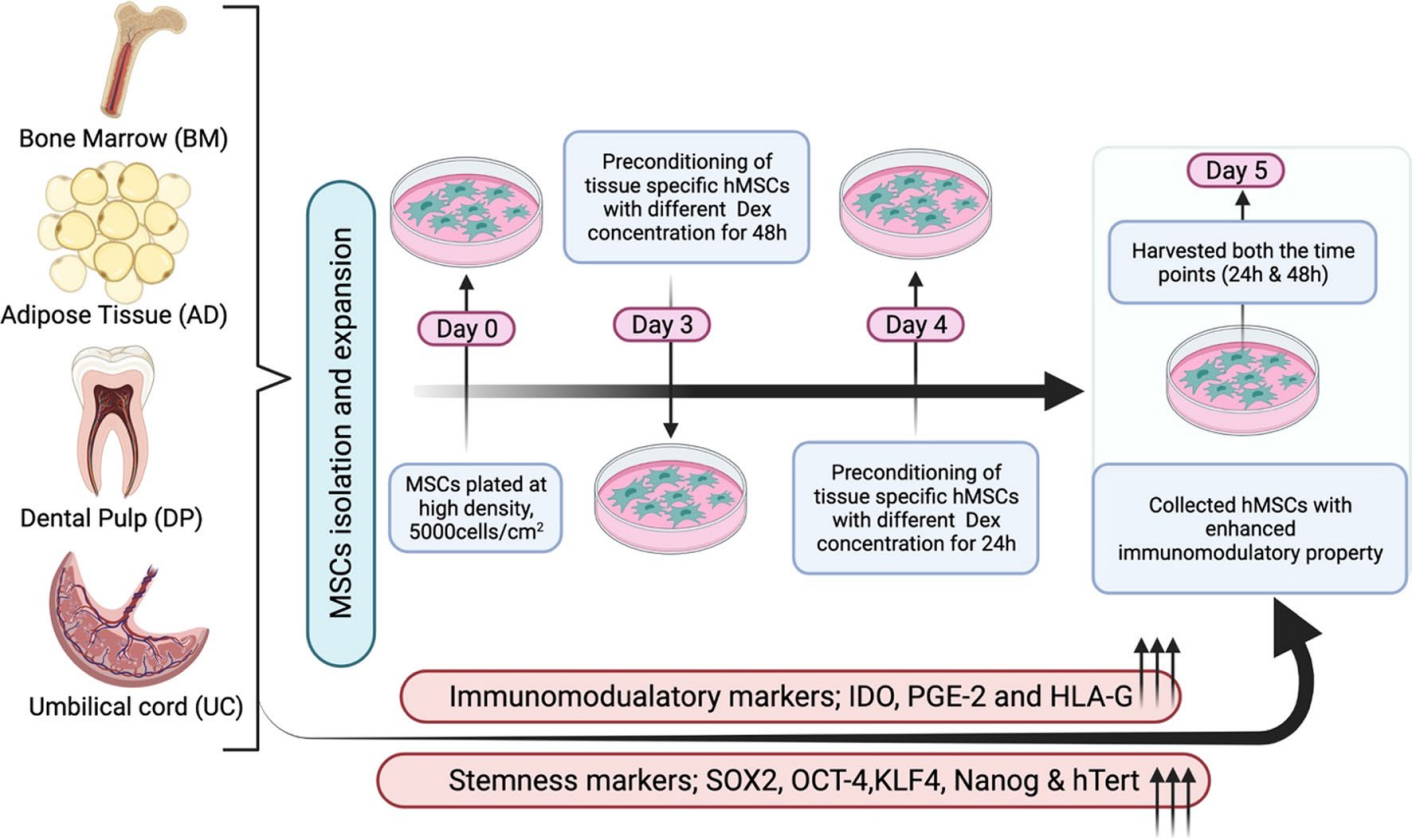

**Supplementary Information:**

The online version contains supplementary material available at 10.1186/s12861-021-00246-4.

## Background

Despite the many advancements in the area of immunosuppressive therapy, graft rejection and graft versus host disease (GvHD) remain the leading cause of post-transplantation mortality [1]. To disparage the risk associated with alloreactivity, immunosuppressive drugs (ISD) like cyclosporine A (CsA), tacrolimus, and mycophenolic acid (MPA) are regular regime after allogeneic transplantation, out of which MPA blocks the calcineurin pathway and results in inhibiting T cell responses. On the other hand, the functional component of mycophenolate mofetil is an anti-proliferative drug that suppresses guanine generation by blocking inosine monophosphate dehydrogenase (IMPDH), respectively [2, 3]*.* Dexamethasone (Dex) is a strong synthetic member of the glucocorticoid class of steroid drugs that act as an anti-inflammatory and immunosuppressant molecule [4]. To achieve systemic inhibition of inflammatory and immune response, the steroids influence various levels of antigen presentation, immune cell proliferation, and cytokine production [5]*.* Therefore, dexamethasone impacts cellular DNA, thereby changing gene transcription [6]. Whereas, use of Dex to treat autoimmune diseases and to prevent the rejection of transplanted organs or tissues in the host, their impact is often followed by detrimental side effects such as nephrotoxicity or osteoporosis, which may diminish their overall benefits [3, 4].

The scientific and clinical interest in human mesenchymal stem cells (hMSCs) has rampant in the past decade, highlighting their role in tissue repair and immunomodulatory properties [7–10]. hMSCs are a plastic adherent heterogeneous population of cells having a fibroblast-like morphology, form colonies in vitro*,* and can differentiate into adipocytes, chondrocytes, and osteocytes [7]. They can be isolated from different organs and tissues, including bone marrow, adipose tissue, dental pulp, muscles, and feto-maternal organs [7]. These cells are known for having low immunogenicity, being able to escape immune cognizance due to negative expression of HLA-class II, and have abilities to express co-stimulatory molecules. hMSCs are also capable of secreting a wide panel of trophic factors and immunomodulatory factors that suppress the local immune response and initiate tissue repair [11, 12]. hMSCs immunoregulatory mechanism is a multifactorial process involving both cells to cell contact and cell-free paracrine signaling. hMSCs secrete a panel of various immunomodulatory factors which aid in reparative and immunosuppressive role [7, 9]. This includes, non-classical HLA-G is one of the influential immunosuppressive molecules, which plays an important role in fetal-maternal tolerance during pregnancy, protecting the fetus from maternal immune cell invasion, and an organ/cell transplantation [13, 14]. Due to the presence of a unique gene promoter at the transcriptional level, the expression of HLA-G is mainly controlled as compared to the classical HLA-class I gene. However, at the post-transcriptional level and after alternate splicing, the primary transcript splits into seven isoforms, out of which four membrane-bound proteins (HLA-G1 to G4) and three are soluble proteins (HLA-G5 to G7). Due to different isoforms, it acts via cell–cell contact or contacts independent action [15].

On the other hand, the molecule generated by Cyclooxygenase (COX-1 and COX-2) enzymes, PGE-2, constitutes another major molecule secreted by hMSCs that exert well-defined actions in a broad spectrum of physiological and pathological settings [16]. It was reported that inflammation conditions led to the upregulation of PGE-2 secretion with it, not only participating in inflammatory response but also initiating proliferation and migration of various cell types [16]. However, there are various possible procedures to prevail hMSCs with enhanced immunosuppressive properties and the potential roles of specific immunomodulatory molecules, which are differentially upregulated in certain culture conditions. Also, various studies have shown that the expression of several molecules implied in hMSCs immunomodulation is regulated by exposure to pro-inflammatory molecules such as IFN- γ, TNF- α, IL-1, and IL-6, etc. [17–19]. Nevertheless, such exposure could also increase hMSCs immunogenicity, impair hMSCs differentiation capacity, and diminish cellular viability [20]. Taking all the above-said attributes into consideration of immunosuppressive drugs, the pre-conditioning treatment approach using immunosuppressive agents with hMSCs may offer a promising alternative strategy, reducing the dosage of immunosuppressive drugs conventionally administered and also improving their efficacy. Therefore, possible caveats must be considered for the use of primed hMSCs, especially for their allogeneic implantation. Therefore, preconditioning hMSCs with steroid, pro-inflammatory cytokine in vitro, before their in vivo administration, is an interesting approach for improving their therapeutic potential.

Reports about the Dex effect on hMSCs gene profile had already been studied and it is widely accepted that Dex is one of the major components for differentiation of hMSCs to osteogenic, Chondrogenic, and Adipogenic [21]. However, available data showed that Dex inhibits the osteogenic differentiation of hMSCs and creates a shift to Adipogenic differentiation in a dose-dependent manner, i.e., at 10^–7^ mol/L. Interestingly, low-dose Dex (10^−8^ mol/L) maintains the cell-surface marker profile of hMSCs over multiple passages [22]. However, much focus has been paid to the effect of Dex on hMSCs proliferation and differentiation. Reports are comparing the differential abilities of tissue specific hMSCs, specifically, placenta-derived hMSCs shown to possess a better proliferative rate and superior engraftment capacity, to share some of the same markers as embryonic stem cells (ESCs) and to present increased immunosuppressive properties [9, 10].

However, the potent immunosuppressive capacity is being considered as a very important feature of hMSCs. In the present study, we have evaluated the effect of Dex in the dose and time-dependent manner on tissue specific hMSCs regenerative properties, immunomodulatory factors, and their in vitro ability to inhibit the proliferation of immune cells.

## Methods

### Isolation and expansion of tissue-specific hMSCs from bone marrow, adipose tissue, dental pulp, and umbilical cord tissue

The study was approved by the Institutional Committee for Stem Cell Research (ICSCR), All India Institute of Medical Sciences (AIIMS), New Delhi, India (Ref No. ICSCR/54/16(R)). All the samples were obtained after taking the donor’s informed consent.

Bone marrow was collected from the donor undergoing the routine medical test procedure in the Department of Hematology, AIIMS, New Delhi. Briefly, BM-MSCs were isolated and cultured as previously described [23, 24]. For Adipose tissue, the sample was collected from the patients undergoing a pre-scheduled surgical procedure in the Department of Pediatric Surgery, AIIMS, New Delhi. The sample was collected in a 5 ml transport vial containing Dulbecco's Modified Eagle Medium (DMEM)-Low Glucose (LG) without FBS with 1% Penicillin (100 U/ml), Streptomycin (100 µg/ml) + Gentamycin (250 µg/ml). The Sample was washed extensively with 1X PBS containing 1% Penicillin (100 U/ml) + Streptomycin (100 µg/ml) + Gentamycin (250 µg/ml) (Gibco, USA). Then explants (~ 2 mm) were carefully placed in a 35 mm culture dish and kept undisturbed, incubated overnight at 37 °C and 5% CO_2_. The next day, as the tissue got adhered to the surface, complete media was added and the medium changed every 3–4 days. When cells started growing and migrating out of the explant and reached 80% confluence, cells were harvested using 0.05% trypsin–EDTA (Invitrogen-Gibco, USA) and transferred into a 60 mm culture dish. Dental pulp derived hMSCs were obtained from the third molar of each individual (16–18 years) who came for orthodontic treatment at the Department of Orthodontics and Dento-Facial Deformities, Centre for Dental Education and Research (CDER), AIIMS, New Delhi. Briefly, DP-MSCs were isolated and cultured as previously described protocol [24, 25].

Umbilical Cord derived MSCs were collected and processed within 24 h of normal or cesarean delivery from the Department of Obstetrics and Gynecology, AIIMS, New Delhi. Briefly, Umbilical Cord was collected in a 50 ml Schott bottle containing 1XPBS with 1% Antibiotics (Penicillin, Streptomycin, and Gentamycin). Upon the arrival of the sample, it was washed extensively with 1XPBS containing 1% Antibiotics. The artery part of the cord was exposed using the sharp surgical blade and it was chopped into a small piece (approx. ~ 2 mm) the exposed jelly part of the cord was placed in a 35 mm culture dish and kept undisturbed. The cultures were incubated overnight at 37 °C and 5% CO_2_ with 1 ml complete medium and changed every three to four days. When cells started growing and migrating out of the explant and reached 80% confluence, cells were harvested using 0.05% trypsin–EDTA (Invitrogen-Gibco, USA) and transferred into a 60 mm culture dish.

Cultures were monitored by phase-contrast microscopy (Olympus, Central Vally, PA) to evaluate the cell morphology and confluency. All assays were performed using tissue-specific hMSCs at passage 3, after their immunophenotypic characterization.

### Pre-conditioning of tissue-specific hMSCs with dex

Stock concentration of Dex (Cat.No. D1756, Sigma, USA) was prepared as per the manufactures protocol. Further, the working concentration was directly prepared in the cell culture medium. Harvested cells were incubated in serum-free medium (control) or in serum-free medium containing various doses of Dex (1000 ng/ml, 2000 ng/ml, 3000 ng/ml) for assessing the effect of these drugs on immunomodulatory molecules (PGE-2, IDO and HLA-G) and stemness markers (Sox-2, Oct-4, Klf-4, Nanog, hTERT) expressed by tissue specific hMSCs. The cells were collected after different exposure times (24 h and 48 h) at all three drug concentrations of Dex. Real-time PCR, Immunofluorescence (IF) staining, Flow cytometry, and ELISA were conducted on the groups, which were collected after 24 h, and 48 h of drug exposure. All the experiment was performed at passage 3.

### Live/dead assay of hMSCs treated with Dex

20,000 cells per well of 24 well plate was plated with tissue specific hMSCs in serum-free culture medium as described previously for 24 h and 48 h with different concentrations of Dex (1000 ng/ml, 2000 ng/ml, 3000 ng/ml). The Live/Dead assay was performed with Calcein- ethidium homodimer dye (Invitrogen, USA). The sample size for each group’s live/dead assay was kept three (n = 3).

### Measurement of metabolic activity by MTT assay of hMSCs treated with dex

The proliferation rate of hMSCs (n = 3) treated without Dex treatment and with Dex treatment was performed. Briefly, 5000 cells per well were seeded in triplicates, and at each termination day (1, 3, 5 and 7) medium was removed and replenish with fresh 180 μl of complete medium and 20 μl of 3-(4, 5-Dimethylthiazol-2-yl)-2,5-diphenyltetrazolium bromide (MTT) (Sigma, USA) reagent was added. The plate was incubated for 3–4 h at 37 °C and 5% CO_2_. Followed by removal of medium and formazan crystals were dissolved with 200 μl of DMSO. The solution was collected in a fresh plate and absorbance were taken at 570 and 660 nm using ELISA reader (BioTek, Germany). The technique was performed as per the previously established protocol [24].

### Scratch assay

To examine whether different concentration and time points of Dex affects the migratory property of tissue specific hMSCs. Tissue specific hMSCs were plated at 50–60% confluency before preconditioning. Culture plates were incubated at 37 °C and 5% CO_2_ until confluency reaches 80–90%. Cells were pre-treated with 1000 ng/ml, 2000 ng/ml and 3000 ng/ml of Dex for 24 h and 48 h in serum-free medium. Followed by PBS wash, a scratch was created using p200 pipet on cells monolayer. Again, culture plates were washed with PBS once and replaced with the DMEM-LG with 10% FBS and 1% Penicillin (100 U/ml) + Streptomycin (100 µg/ml). At 0 h, 12 h, 24 h, the scratch area was imaged using phase-contrast microscopy (Olympus, Central Vally, PA) with a 4X magnification lens. The analysis of open area was performed using Image J software. The experiment was performed thrice for all the tissue-specific hMSCs.

### Trilineage differentiation

Tissue specific hMSCs were characterized by differentiating cells into osteogenic, chondrogenic and Adipogenic lineages according to the induction protocols described in our earlier published research article [23, 24].

### Immunophenotyping

At passage 3, hMSCs were characterized using monoclonal antibodies specific for CD105-APC, CD73-PE, CD29-FITC, CD90-PerCp-Cy5.5, HLA-ABC-APC, HLA-DR-FITC, CD34/45-PE/FITC (BD Pharmingen, France). 50,000 cells were incubated with the respective primary mAb or isotype‐matched control antibody for 40 min in dark. Cells were washed with 1X PBS and analyzed by flow cytometry (BD-LSR-II, San Jose, CA). For HLA-G Flow cytometry studies, the mouse anti-HLA-G1/HLA-G5 MEMG/9 PE antibody (Exbio, Praha, Czech Republic) was used at 1:200 final concentration for 75,000 cells and incubated for 40 min in the dark. For analysis, isotype controls were included. The average of HLA-G was calculated value from 5 donors for all the tissue-specific hMSCs. Acquisition and data analysis were performed using flow cytometry (BD Bioscience) and FACS Diva Software Version 6.2.

### Quantitative real-time reverse transcriptase-polymerase chain reaction (qRT-PCR)

For the isolation of total RNA from cultured tissue-specific hMSCs, the respective T-25 flasks were washed using PBS for the removal of any existing debris or serum. The cells were then trypsinized using Trypsin–EDTA, mixed with complete media, and pellet down at 300 g (Beckman Coulter, California, USA) for 5 min. The cells were transferred into microcentrifuge tubes (MCT) and were then lysed using TRI reagent (Molecular Research Centre, Ohio, USA), 1 ml/ 1 × 10^6^ cells. The total RNA was prepared according to the phenol–chloroform extraction method. The concentration and optical density (OD) of samples were recorded using a Nanophotometer (Implen, Germany). Reverse Transcriptase PCR: cDNA was prepared, using 2 µg/µl of the RNA samples from tissue-specific hMSCs and JEG-3, HeLa cell lines by Reverse transcriptase (RT) enzyme (Promega, USA). Optimization of cDNA using GAPDH: Glyceraldehyde—3 phosphate dehydrogenase (GAPDH) was used in the PCR setup as the housekeeping gene, for the optimization of the prepared cDNA samples of tissue-specific hMSCs, JEG-3 and, HeLa. qPCR was performed in duplicates using SYBR green Master Mix according to the manufacturer’s instruction (Kappa, USA). We calculated the average fold change for PGE-2, IDO and HLA-G value from 5 donors. However, for stemness markers studies, the average value of 3 donors were considered for tissue-specific hMSCs in duplicate using an equation of the standard curve. List of primers (Table 1).

**Table 1:** List of primers

S. Nos	Gene	Sequence		Tm	Company
1	OCT-4	FP	5′-AGCGAACCAGTATCGAGAAC-3′	59.6	Sigma
		RP	5′-TTACAGAACCACACTCGGAC-3′		
2	Sox-2	FP	5′-AGCTACAGCATGATGCAGGA-3′	59.6	Sigma
		RP	5′-GGTCATGGAGTTGTACTGCA-3′		
3	Nanog	FP	5′-TGAACCTCAGCTACAAACAG-3′	59.6	Sigma
		RP	5′-TGGTGGTAGGAAGAGTAAAG-3′		
4	Klf-4	FP	5′-TCTCAAGGCACACCTGCGAA-3′	59.6	Sigma
		RP	5′-TAGTGCCTGGTCAGTTCATC-3′		
5	hTERT	FP	5′-ACCAAGCATTCCTGCTCAAGCTG-3′	55.7	Eurofin
		RP	5′-CGGCAGGTGTGCTGGACACTC-3′		
6	HLA-G	FP	5′-CTGACCGAGACCTGGGCGGGCT-3′	60.8	Sigma
		RP	5′-GGCTCCATCCTCGGACACGCCGA-3′		
7	IDO	FP	5′-CCTGAGGAGCTACCATCTGC-3′	50.7	Sigma
		RP	5′-TCAGTGCCTCCAGTTCCTTT-3′		
8	PGE-2	FP	5′-ACTCTGGCTAGACAGCGTAA-3′	62.8	Sigma
		RP	5′-ACCGTAGATGCTCAGGGAC-3′		
9	GAPDH	FP	5′-GAC AAG CTT CCC GTT CTC AG-3′	55	Sigma
		RP	5′-GAC AAG CTT CCC GTT CTC AG-3′		

### ELISA for PGE-2 and HLA-G

Tissue specific hMSCs, pre-treated with different concentrations of Dex (1000 ng/ml, 2000 ng/ml, 3000 ng/ml) for 24 h and 48 h, were seeded in 6 well plates at 50,000 cells/well and cultured for respective time and dose concentration in 2 ml medium without FBS. Culture supernatant was then collected and the concentration of PGE-2 determined by ELISA (Cayman, USA), whereas HLA-G has been determined by ELISA kit (Biovender, USA). Each group of different Dex concentrations was performed in duplicates (n = 3) for all tissue specific hMSCs.

#### IDO activity

The biological activity of IDO was calculated by measuring the level of kynurenine in supernatant collected from different preconditioned tissue-specific hMSCs. Briefly, 100 μl of collected cell culture supernatant were added to the Eppendorf tube and 50 μl of 30% trichloroacetic acid (Sigma, USA) were added, the tube was vortexed and centrifuged at 8000 g for 5 min. Then, 75 μl of the supernatant was transferred with an equal volume of Ehrlich reagent (100 mg p-dimethyl benzaldehyde in 5 ml glacial acetic acid) (Sigma, USA) to a 96-well microtiter plate and recorded the absorbance at 490 nm [26]. Experiment was performed at least in three independent setups.

#### Immunosuppression activity of tissues specific on peripheral blood mononuclear cells (PBMCs)

The study was approved by the Institutional Ethics Committee (IEC) (Ref No. IECPG-345/07.09.2017, (RT-6/29.11.2017). All the samples were obtained after taking the donor’s informed consent. Human Peripheral blood mononuclear cells (PBMCs) were isolated by Ficoll-Paque (Axis-Shield; Oslo, Norway) density gradient centrifugation from blood donated by healthy volunteers. Phytohemagglutinin A (PHA (Sigma, USA); 35 µg/mL) was used to stimulate the activation of human peripheral blood mononuclear cells (PBMCs) before co-culture. For co-culture experiments, hMSCs were treated with Mitomycin C (Sigma, USA); 15 µg/ml and co-cultured (1 × 10^4^ cells/well) with PHA activated hPBMCs (5 × 10^4^ cells/well) in 1:5 ratio in RPMI-1640 medium (Gibco, USA) containing 10% FBS for 3 days in 96-well plates (Costar, USA). Proliferation of hPBMCs were assessed by MTS assay. The 200 μl cell culture supernatant containing hPBMCs were collected in pre-labelled 0.6 ml Eppendorf tube and 20 μl of MTS reagent (Promega, USA) was added in each tube, followed by incubation for 3 h at 37 °C and 5% CO_2_. Afterwards, tubes were centrifuged at 300 g for 5 min. 200 μl of supernatant was collected from each tube and transferred into the fresh 96 well plate. The absorbance was taken at 490 nm using ELISA reader (Biotek, Germany). Lastly, % decrease was calculated by calculating difference between positive control (Activated PBMCs) and test group (MLR). Then; divided the decrease by the positive control and multiplied the answer by 100.

#### Statistical analysis

Statistical analysis was performed using Two way-ANOVA, post-test Tukey and *t*-test in GraphPad Instant software (GraphPad Software, Inc.).

## Results

Tissue specific hMSCs were isolated, expanded and characterized according to ISCT guidelines [27]. It was observed that all hMSCs showed plastic adherence and spindle shaped morphology. Surface marker profiling shows > 95% positivity for CD105, CD90, CD73, CD29, HLA-Class I and negative for HLA-class II, CD34/45. Further, all the tissue specific hMSCs were induced to trilineage differentiation i.e., Osteocytes, Adipocytes and Chondrocytes. Where osteocytes differentiation was confirmed using alizarin red staining representing mineralization of cells, where oil red ‘o’ staining reveals oil droplets formation and confirms the adipocytes differentiation and chondrocytes differentiation was confirmed using Alcian blue staining (Additional file 1: Fig. S1).

### Effect of dexamethasone on basic characteristic properties of tissue specific hMSCs

Upon preconditioning of hMSCs with different concentrations of Dex at different time points, tissue specific hMSCs did not show any dose and time-dependent response pattern. The range of studied dose concentrations were taken from the available literature [28, 29]. The morphology of BM-MSCs, AD-MSCs, DP-MSCs, and UC-MSCs is presented. They maintained their spindle shape, elongated morphology even at different concentrations and time points of Dex treatment (Additional file 1: Fig. S2).

**Fig. 1:**
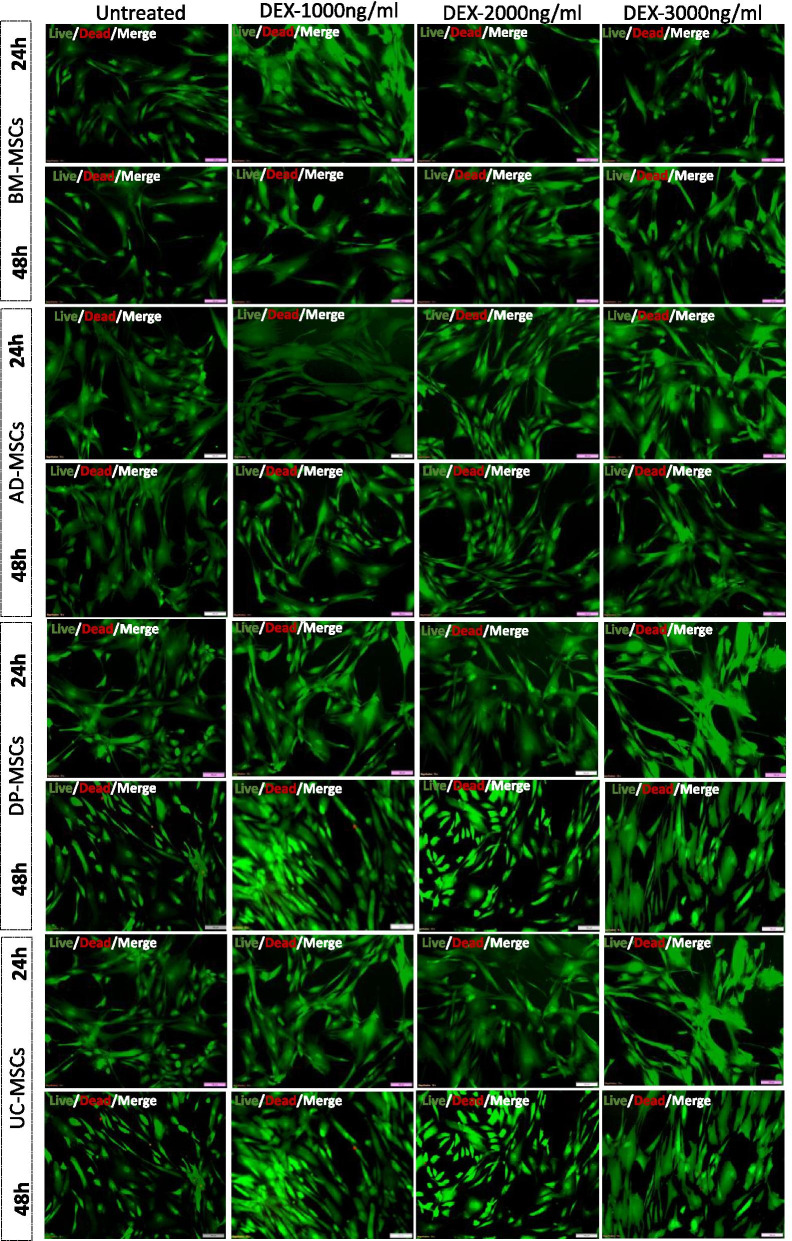
Cytotoxicity of Dex at different dose concentrations and time points; A representative image showing the Live/Dead Staining upon pre-conditioning with different concentrations of Dex on tissue specific hMSCs. Scale bar 100 µm

The live/dead staining was performed to show Dex cytotoxicity (Fig. 1). The green signal shows all live cells whereas the red signal represents the dead cells. However, 1000 ng/ml, 2000 ng/ml, and 3000 ng/ml concentration at different time points of 24 h and 48 h did not show any cytotoxic effect on all the tissue specific hMSCs.

**Fig. 2:**
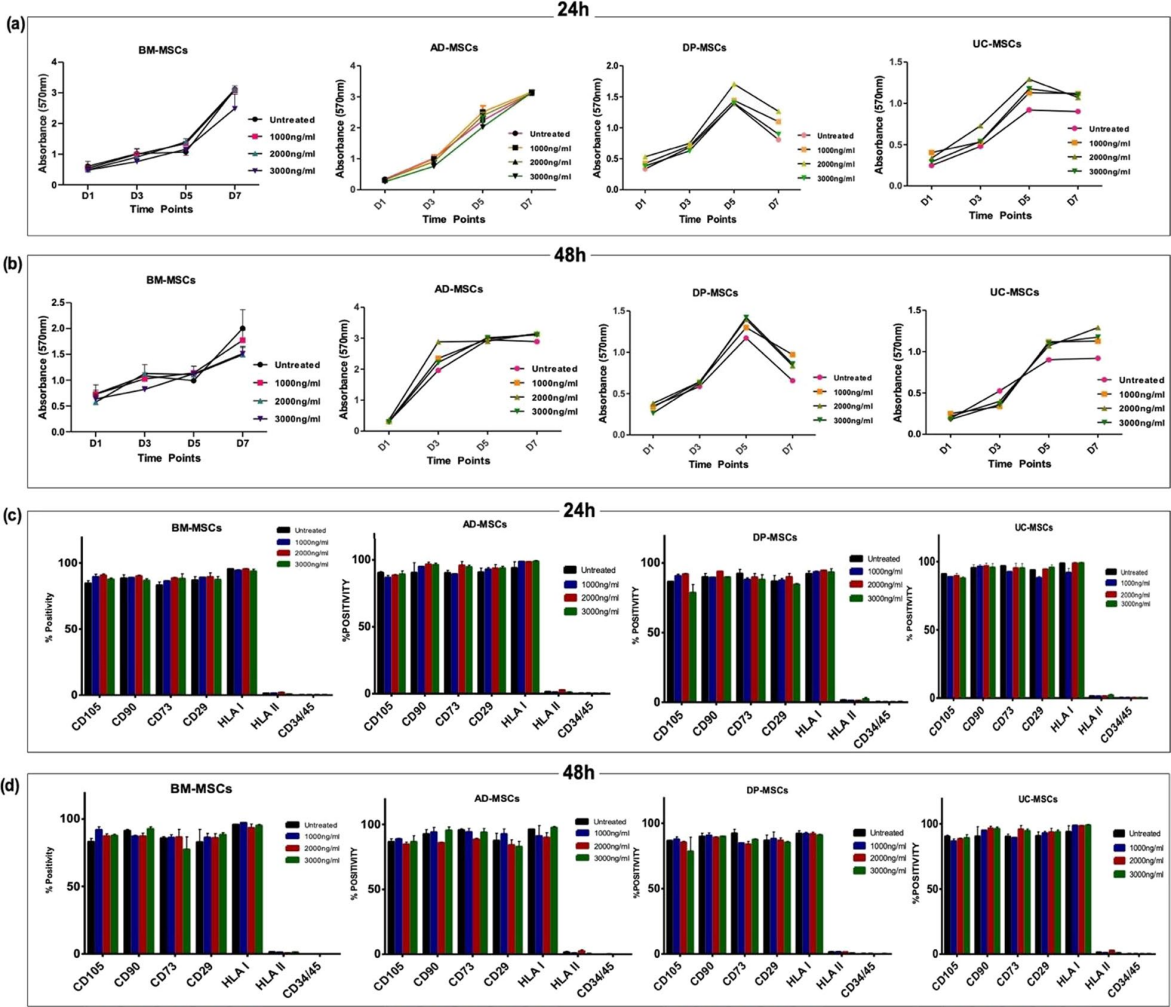
Effect of Dex on cell proliferation and surface marker profiling of tissue specific hMSCs (**a**, **b**) A representative line graph shows cell growth capacity and (**c**, **d**) surface marker expression of tissue specific hMSCs upon pre-conditioning of different concentrations of Dex on tissue specific hMSCs. Positive marker CD 105, CD 90, CD 73, CD 29, and HLA-class I showed above 95% positivity in all study groups and negative markers HLA-Class II and CD 34/45 did not show any expression at all the study groups. Data from three donors of each tissue-specific hMSCs

MTT assay was performed to evaluate the metabolic activity of the cells after preconditioning with Dex at different concentrations and time points. hMSCs were analyzed for Day 1, Day 3, Day 5 and Day 7 (Fig. 2a, b). The treated and untreated cells showed a typical sigmoid curve for cell metabolic activity, whereas at Day 1 and Day 3 all cells showed the highest proliferation followed by a decreasing trend on Day 7, due to high cell confluency and contact inhibition. Among all the studied groups, no significant difference was observed in Dex treated hMSCs compared to untreated hMSCs.

Furthermore, immunophenotyping was performed for tissue-specific hMSCs upon treatment with different concentrations at various time points. CD105, CD90, CD73, CD29, and HLA-Class I showed > 95% expression for all the treated and untreated tissue-specific hMSCs, whereas HLA-Class II and CD34/45 showed negative expression for treated as well as untreated tissue-specific hMSCs (Fig. 2c, d).

Various alluring features of hMSCs makes them an ideal candidate to be used for the treatment of several diseases, yet the therapeutic efficacy of hMSCs is unpredictable due to change in inflammation microenvironment and effecting the homing. However, a scratch assay was performed to determine the effect of Dex on the migratory property of hMSCs. The open area was evaluated at 0 h, 12 h, and 24 h in tissue-specific hMSCs (Additional file 1: Fig. S3).

**Fig. 3:**
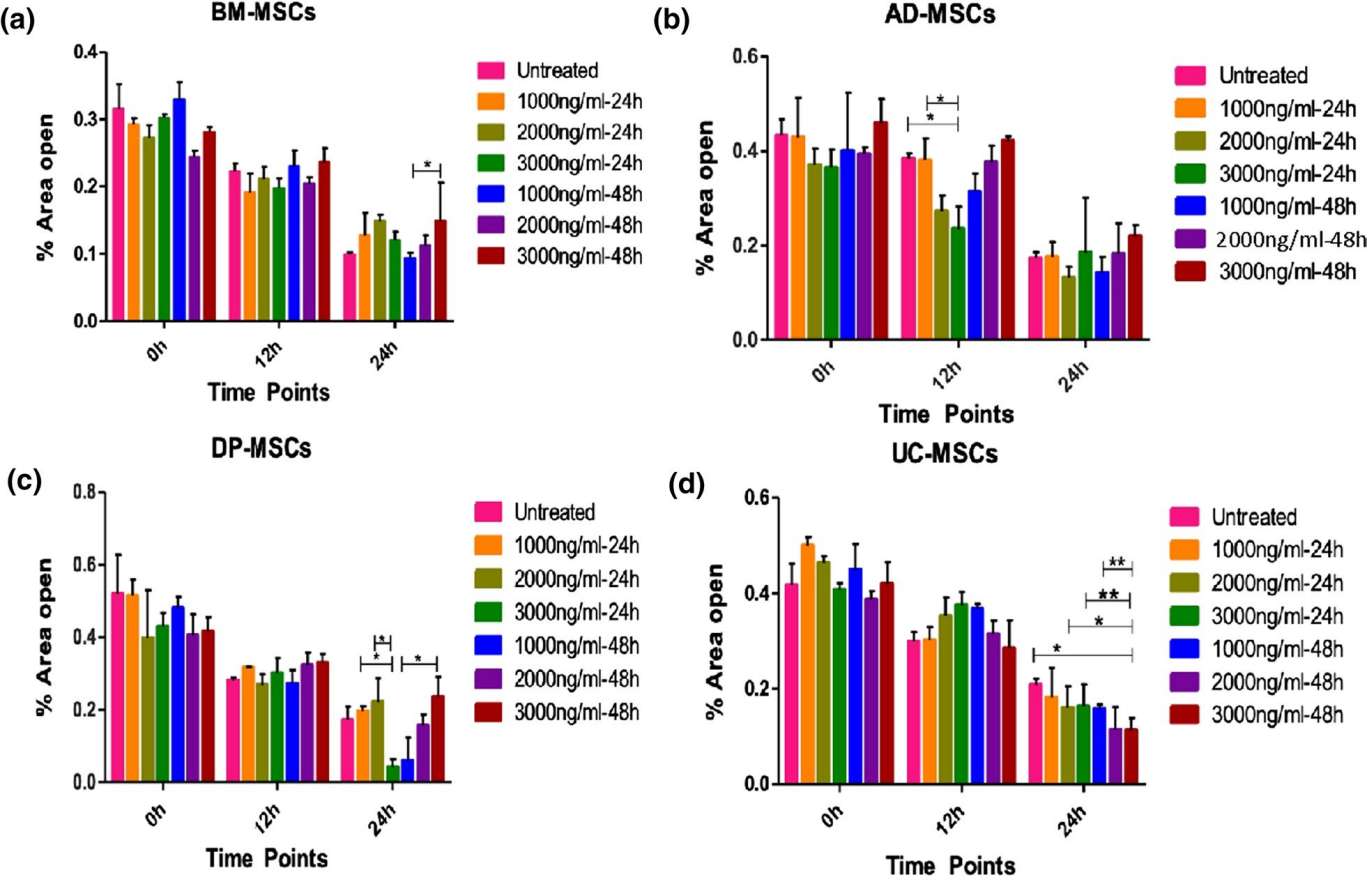
Effect of different concentration of Dex on the migratory property of tissue specific hMSCs; The representative bar graphs of tissue specific hMSCs shows the scratch area at 0 h, 12 h, and 24 h after preconditioning with Dex in the dose and time-dependent manner. **a** A bar graph represents the % area open from 0 to 24 h; BM-MSCs shows the maximum closed area at 1000 ng/ml 48 h, **b** AD-MSCs shows the almost similar response at all the dose concentration and time points; DP-MSCs showed significant closed area at 3000 ng/ml-24 h, 1000 ng/ml 48 h whereas UC-MSCs shows the response at 3000 ng/ml 48 h in all the study groups. Data from three donors and shown as mean ± SD; **p < 0.01, *p < 0.05

**Fig. 4:**
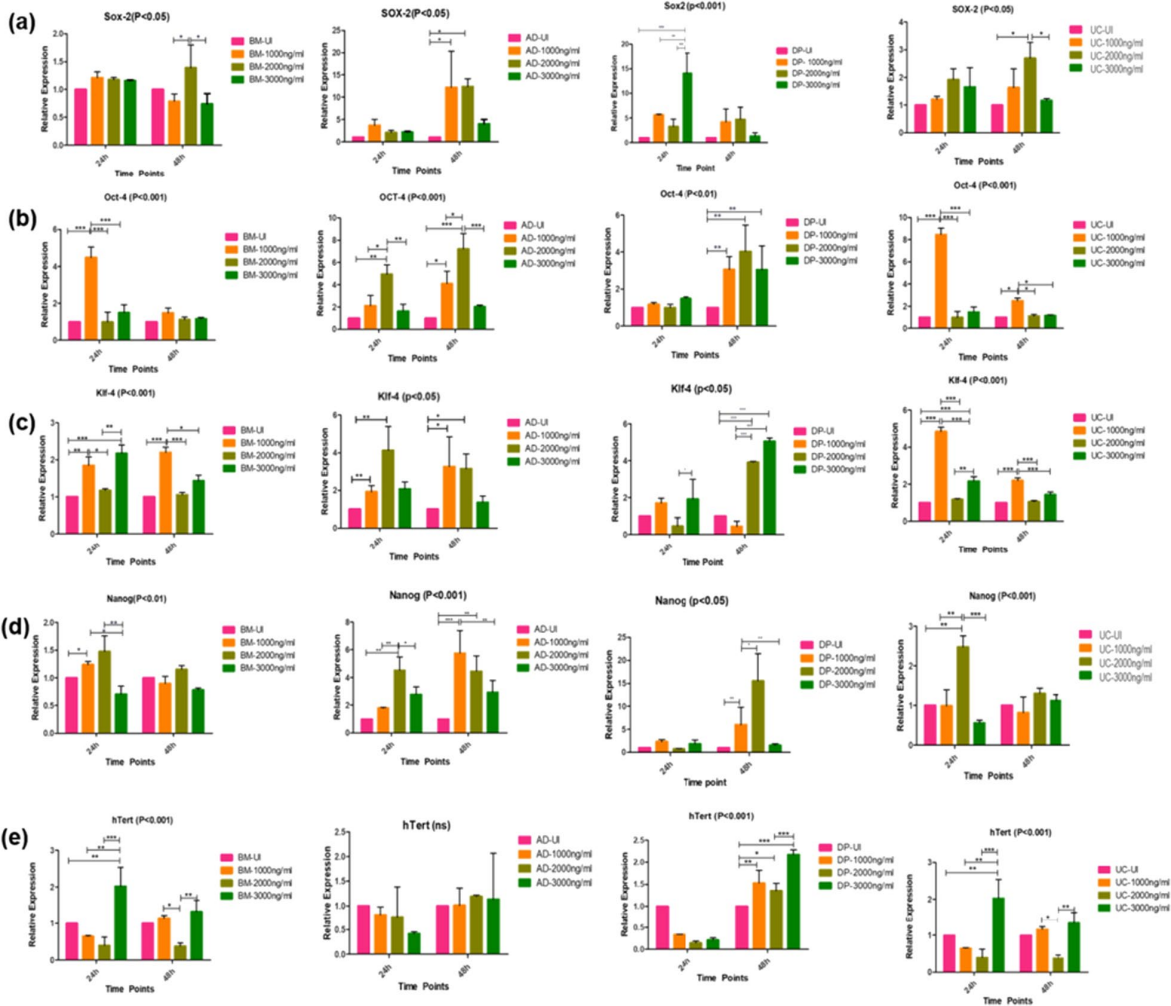
A representative bar graphs shows relative expression level of stemness markers **a** Sox-2, **b** Oct-4, **c** Klf-4, **d** Nanog, **e** hTERT in tissue specific hMSCs at different Dex concentration and time points**.** Data from three donors and shown as mean ± SD; ***p < 0.001, **p < 0.01, *p < 0.05

Dex preconditioning shows the upregulated migratory property of hMSCs, specifically BM-MSCs, and DP-MSCs showed the maximum area closure at 1000 ng/ml for 48 h. However, AD-MSCs did not show any significant change in percentage closed area but UC-MSCs showed maximum closed area at 3000 ng/ml, 48 h (Fig. 3). In order to study the stemness of tissue specific hMSCs after preconditioning with different concentrations of Dex at different time points, we analyzed the signature gene expression of (Sox-2, Oct-4, Nanog, Klf-4, and hTert) transcriptional and translational regulatory network in tissue specific hMSCs, compared to untreated hMSCs. In the treatment group, DP-MSCs and AD-MSCs displayed the highest expression level of stemness markers followed by UC-MSCs and BM-MSCs (Fig. 4). Moreover, Klf-4, Nanog and Sox-2 showed a strong upregulation in DP-MSCs and AD-MSCs at 48 h of all different concentrations mostly at 2000 ng/ml. However, telomerase gene (hTert) expression is similar in DP-MSCs and AD-MSCs but the lowest was observed in BM-MSCs and UC-MSCs. To further confirms the protein level expression was assessed at 48 h with all the dose concentrations via immunofluorescence staining for SOX-2, Nanog and OCT-4, (Additional file 1: Figs. S4, S5 and S6).

### Effect of dexamethasone in the dose and time-dependent manner on immunomodulatory factors (IMF)

Dex is an anti-inflammatory steroid. Treating with either Dex or hMSCs for allogenic transplants are well established conventional lines of treatment; the effect of different concentrations of Dex on hMSCs immunomodulatory property. However, it is unknown. Therefore, to examine the effect of this interaction, BM-MSCs, AD-MSCs, DP-MSCs and UC-MSCs were pre-conditioned with 1000 ng/ml, 2000 ng/ml, and 3000 ng/ml of Dex for 24 h and 48 h and then, PGE-2, IDO and HLA-G1/G5 were assessed at both gene level and protein level.

#### qRT-PCR assessment of PGE-2, IDO and HLA-G1/G5

**Fig. 5:**
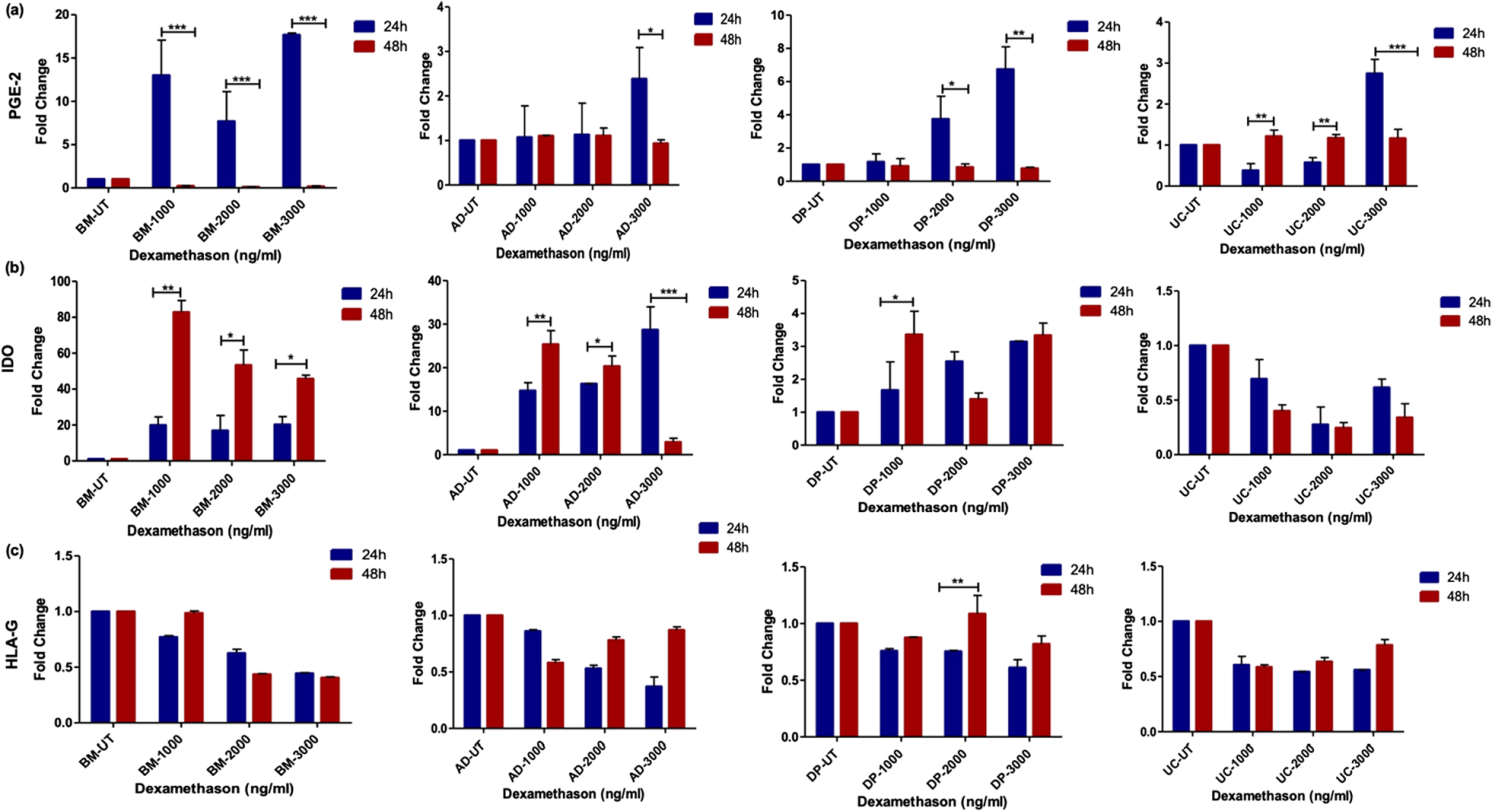
qRT-PCR assessment of PGE-2, IDO and panHLA-G. All the tissue specific hMSCs were treated with Dex at different dose concentration and time points **a** Fold change of PGE-2; Shows the significant upregulation in BM-MSCs and DP-MSCs at 24 h, whereas AD-MSCs and UC-MSCs did not respond for Dex in terms of PGE-2 expression, **b** Fold change of IDO; shows the significant upregulation in BM-MSC and AD-MSCs at 48 h, similarly, DP-MSCs shows significant upregulation at 1000 ng/ml for 48 h, whereas, UC-MSCs did not shows any significant upregulation, **c** Fold change of HLA-G; HLA-G did not shows any significant upregulation in all the dose and time point. Data from five donors and shown as mean ± SD; ***p < 0.001, **p < 0.01, *p < 0.05

Relative expression of PGE-2 in tissue specific hMSCs; BM-MSCs showed a 15-fold change at 1000 ng/ml ≅ 3000 ng/ml at 24 h (*p* < 0.001) an enhanced response towards Dex, whereas it did not show any response at 48 h. Here, DP-MSCs ranked second among the other hMSCs, showed a nine-fold change at 2000 ng/ml ≅ 3000 ng/ml for 24 h (*p* < 0.01), and no response at 48 h. However, AD-MSCs and UC-MSCs showed a minimal response to different concentrations and time points (Fig. 5a). The relative expression of IDO in tissue specific hMSCs was evaluated where BM-MSCs (80-fold change) (*p* < 0.001) showed highest fold change followed by AD-MSCs (40-fold change) (*p* < 0.001) and DP-MSCs (fivefold change) (*p* < 0.05) at 1000 ng/ml at 48 h, 3000 ng/ml for 48 h and 1000 ng/ml for 24 h respectively. However, UC-MSCs did not showed any change in expression level of IDO at any preconditioning (Fig. 5b). Whereas, relative expression of panHLA-G in tissue specific hMSCs did not show any increase at the gene level in either of the tissue specific hMSCs upon pre-treatment with different concentrations of Dex and time points (Fig. 5c).

#### Protein level assessment of PGE-2 and HLA-G1/G5

##### Surface and intracellular expression of HLA-G assessment in Dex treated tissue specific hMSCs

**Fig. 6:**
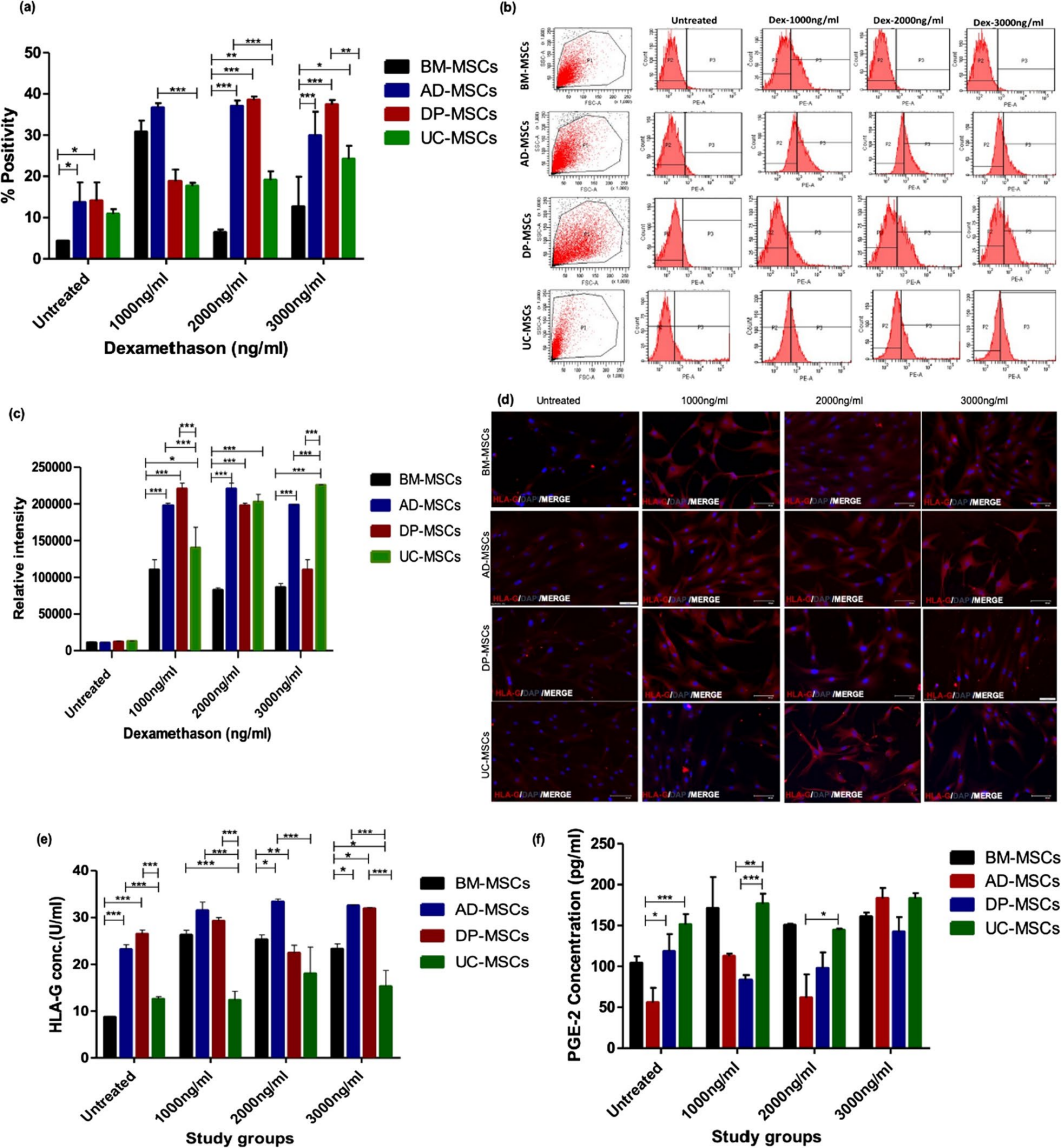
Protein level assessment of HLA-G1/G5 and PGE-2 after 24 h of preconditioning: **a** A bar graph represents the % Positivity of BM-MSCs responds at 1000 ng/ml, AD and DP-MSCs responds at 2000 ng/ml whereas DP-MSCs shows significant upregulation at 3000 ng/ml and UC-MSCs shows significant upregulation at 3000 ng/ml, **b** Pictographs represents the histograms for tissue specific hMSCs using BD FACs Diva™ software. Intracellular expression of HLA-G in tissue specific hMSCs; **c** and **d** A pictograph and bar graph represent the relative intensity of intracellular HLA-G expression in tissue specific hMSCs. ELISA data for soluble HLA-G5 and PGE-2 in cell culture supernatant at 24 h of Dex preconditioning; **e** A bar graph represents the BM-MSC responds at 1000 ng/ml, AD-MSCs responds at 2000 ng/ml whereas AD and DP-MSCs showed significant upregulation at 3000 ng/ml and UC-MSCs did not showed significant secretion of sHLA-G, **f** Bar graph represents that BM-MSCs shows highest expression level at 1000 ng/ml, AD-MSCs at 3000 ng/ml, DP-MSCs at 3000 ng/ml whereas UC-MSCs showed significant upregulation at 1000 ng/ml. Data from three donors in duplicates and shown as mean ± SD; ***p < 0.001, **p < 0.01, *p < 0.05. Scale bar 100 µm

As HLA-G did not show any response at the gene level, therefore, 24 h was selected as optimum time point to assess surface and intracellular expression of HLA-G1 and HLA-G1/G5 at all studied Dex concentration. BM-MSC responded at 1000 ng/ml showing 30 ± 10% cells positive for HLA-G1, AD and DP-MSCs responded at 2000 ng/ml showing 25 ± 10% positive expression whereas DP-MSCs and UC-MSCs showed significant upregulation at 3000 ng/ml of Dex concentration (Fig. 6a, b).

To investigate the intracellular expression of HLA-G1/G5 in tissue-specific hMSCs, immunofluorescence staining was performed. It was observed that BM-MSCs respond at 1000 ng/ml, AD and DP-MSCs responded at 2000 ng/ml whereas DP-MSCs showed significant up-regulation at 3000 ng/ml and UC-MSCs shows significant up-regulation at 3000 ng/ml. Altogether, BM-MSCs, AD-MSCs, and DP-MSCs showed a comparable response to Dex at 1000 ng/ml and 2000 ng/ml. Whereas, UC-MSCs showed a significant response at 3000 ng/ml compared to untreated UC-MSCs (Fig. 6c, d).

##### ELISA for assessment of PGE-2 and HLA-G5 in cell culture supernatant of treated and untreated tissue-specific hMSCs

Tissue specific hMSCs were preconditioned with different Dex concentrations for 24 h as gene level studies showed significant upregulation of PGE-2 at this time point. The culture supernatant was collected and proceeded to perform ELISA for HLA-G5 and PGE-2. Where, BM-MSC responded at 1000 ng/ml, AD-MSCs responded at 2000 ng/ml whereas AD and DP-MSCs showed significant up-regulation at 3000 ng/ml. However, UC-MSCs did not show significant secretion of soluble HLA-G (Fig. 6e and f). Moreover, BM-MSCs showed 210 pg/ml (*p* < 0.001), DP-MSCs showed 150 pg/ml (*p* < 0.001), a significant amount of PGE-2 whereas AD-MSCs and UC-MSCs showed 50 pg/ml (*p* < 0.05), of PGE-2 level at 2000 ng/ml of Dex treatment.

##### IDO activity in cultured supernatant of treated and untreated tissue-specific hMSCs

**Fig. 7:**
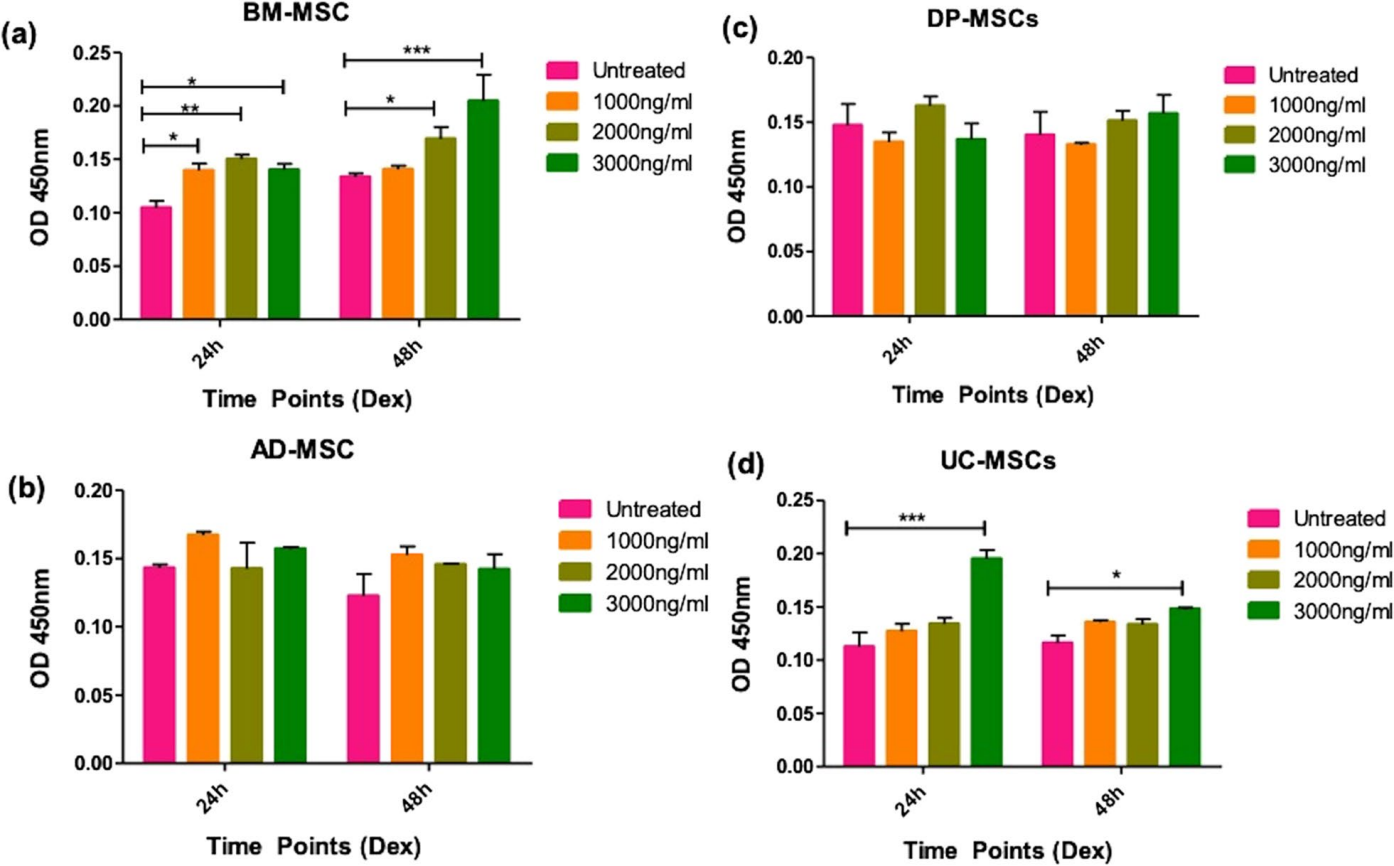
IDO activity of tissue specific hMSCs after pre-conditioning with different Dex concentration at 24 h and 48 h; **a** The bar graph represents that BM-MSCs after preconditioning with 3000 ng/ml at 48 h shows significant upregulation among the other preconditioning combinations. **b** and **c** The bar graph represents that AD-MSCs and DP-MSCs shows similar response to Dex preconditioning compared to untreated hMSCs. **d** The bar graph represents that UC-MSCs after preconditioning with 3000 ng/ml for 24 h shows significant upregulation. Data from three donors in duplicates and shown as mean ± SD; ***p < 0.001, **p < 0.01, *p < 0.05

IDO activity was determined by measuring the level of kynurenine in supernatant collected at 24 h and 48 h from untreated and Dex pre-conditioned tissue specific hMSCs. Pre-conditioned BM-MSCs and UC-MSCs shows significant amount of upregulation at 3000 ng/ml for 48 h. On contrary, AD-MSCs, DP-MSCs did not shows any significant IDO activity after Dex pre-conditioning (Fig. 7).

#### Differential immune-suppressive ability of tissue specific hMSCs

**Fig. 8:**
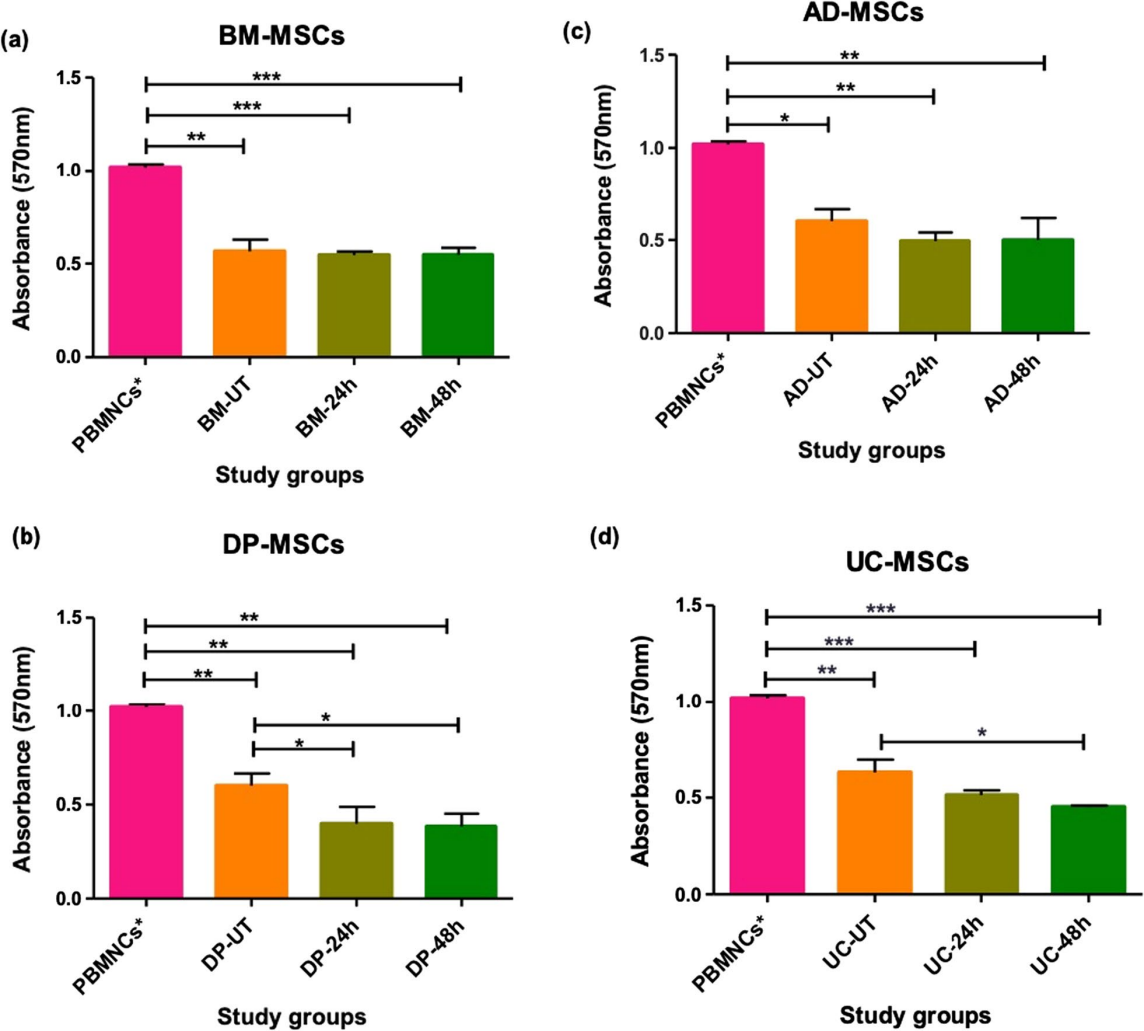
Immunosuppressive activity of tissue-specific hMSCs in allogeneic condition after pre and post-preconditioning with Dex; **a** and **c** The bar graph represents that 48 h Dex-pretreated BM-MSCs and AD-MSCs shows the significant ability to inhibit the PBMCs proliferation with respect to activated PBMCs, **b** and **d** the bar graph represents that 48 h Dex-pretreated DP-MSCs and UC-MSCs shows significant inhibition compared to untreated hMSCs and activated hMSCs. Data from three donors in duplicates and shown as mean ± SD; ***p < 0.001, **p < 0.01, *p < 0.05. Note: PBMCs* (PHA activated PBMCs)

The immuno-suppressive capacity of these hMSCs was assessed under untreated and pre-conditioning with 3000 ng/ml for 24 h and 48 h via one-way mixed lymphocyte reaction. The culture condition was selected as all the tissue specific hMSCs showed significant response. The inhibition of proliferation of peripheral blood mononuclear cells (PBMCs) was observed in each hMSCs groups, against the positive control i.e., PHA activated PBMCs (PBMCs*) (Fig. 8). The percentage decrease of PBMCs suppression was calculated (Table 2). It was observed that untreated BM-MSCs showed 44.5% decrease whereas pre-conditioned BM-MSCs showed similar decrease i.e., 46.32%. AD-MSCs showed similar response as BM-MSCs i.e., untreated group showed 41.11% decrease and pre-conditioned group showed 51.03% decrease. Unlike BM-MSC and AD-MSCs, DP-MSCs showed significant decrease at 3000 ng/ml preconditioning for 24 h and 48 h, i.e., 60.94% and 62.41% decrease respectively. Similarly, UC-MSCs showed significant decrease at 3000 ng/ for 48 h preconditioning, i.e., 55.64%. Overall, DP-MSCs and UC-MSCs showed significant immune suppression as compared to AD-MSCs and BM-MSCs.

**Table 2:** Percentage decrease in the proliferation of immune cells

S. Nos	Culture conditions	Average OD (n = 5)	% Decrease
1	PBMCs	1.0195	
2	MLR-BM-UT	0.5655	44.5
3	MLR-BM-24	0.5455	47.79
4	MLR-BM-48 h	0.547	46.32
5	MLR-AD-UT	0.600	41.11
6	MLR-AD-24	0.4945	51.52
7	MLR-AD-48 h	0.499	51.03
8	MLR-DP-UT	0.612	37.34
9	MLR-DP-24	0.398	60.94
10	MLR-DP-48 h	0.383	62.41
11	MLR-UC-UT	0.630	38.17
12	MLR-UC-24	0.513	49.65
13	MLR-UC-48 h	0.452	55.64

## Discussion

Mesenchymal stem cells are multipotent, non-hematopoietic cells with different tissue origins. They have become a promising candidate for the treatment of many autoimmune and allogeneic transplant conditions due to their immunoregulatory, anti-inflammatory, and pro-regenerative properties. hMSCs can be isolated from different tissue sources such as adipose, umbilical cord, dental pulp, bone marrow, etc. and depending upon the tissue origin they display varying features in vitro and in vivo [30, 31]. So far, BM-MSCs and AD-MSCs are most frequently being used for clinical trials involving degenerative and autoimmune diseases. Till now, it has been observed that all the tissue specific hMSCs display similar basic characteristic properties but it is still unclear which tissue source has a better functionality at the time of inflammation. Inflammatory microenvironments could also produce a rise in hMSCs immunogenicity, which might pose adverse safety and efficacy implications for their allogeneic use [32, 33]. Therefore, maintenance of low immunogenicity even under inflammatory stress is an important factor to consider for prospecting safe hMSCs’ allogeneic transplantation. However, the need of the hour is to have a different approach to precondition the hMSCs for inflammatory microenvironment. Several clinical studies have shown that various immunosuppressive drugs and hMSCs are being co-administered as a line of treatment for autoimmune diseases, due to which few of hMSCs properties are being compromised such as homing and cytoskeleton changes [34]. To the best of our knowledge, the present study is the first report to compare the effect of Dex pre-treatment on tissue specific hMSCs for its therapeutic potential, by evaluating a range of their basic characteristics. Herein, we demonstrated that this pre-treatment approach is highly effective in augmenting the immunotherapeutic function of hMSCs in terms of immunomodulation. In vivo, the concentration of glucocorticoids is markedly upregulated in maternal plasma and amniotic fluid near the fetus, during the pre-term or expected time of parturition [18]. They promote HLA-G expression and Th2 cytokine secretion profile, thereby preventing the semi-allogeneic fetus from allograft rejection by the maternal immune system [35]. To mimic this condition, we focused on examining the direct effect of Dex on hMSCs, without interfering with hMSCs native properties and offering an improvised product for therapeutic purposes. In this regard, a time-dependent study of different concentrations of Dex was performed to understand its effects on immunosuppressive properties [35]. It was observed that Dex did not show any effect on the cellular morphology at either of the concentrations (1000, 2000, 3000 ng/ml) and is non-cytotoxic to all the tissue specific hMSCs. Cody et al.reported the differential cytotoxicity of various corticosteroids on AD-MSCs, with Dex showing the least toxic effects at 4 mg/ml for 24 h as compared to other tested drugs [36]. Whereas, in the present study maximum of 3000 ng/ml for 48 h was studied and showed no toxicity.

It was observed that Dex treatment did not alter their surface marker profile, as per the criteria set by ISCT guidelines, along with metabolic activity. Notably, In Hwan song and Seong Yong reported that Dex treatment for 3 weeks influences the proliferation rate of hMSCs in long term culture by suppression of apoptosis [37]. Whereas, another study highlighted that 10^–8^ M dose of Dex maintains proliferative potential, stemness and non-differential potential of cells [22]. As a matter of fact, Dex is known to be an important inducer for osteogenic and Adipogenic lineages [20]. In the present study, it was observed that Dex can elicit stemness markers in a dose–response manner. Whereas, higher the dose of Dex can treat them towards differentiation. However, it is favorable to use the low concentration of Dex to enhance stemness properties of hMSCs. Moreover, this study has allowed to maximize the stemness of tissue specific hMSCs at low concentration of Dex without any maximum manipulation.

Interestingly, it is the first report where scratch assay showed that Dex preconditioning has upregulated the migratory property of tissues specific hMSCs. On contrary, Schneider et al. reported that treatment of hMSCs from human term chorionic membranes with 10 µM Dex for 24 h resulted in elevated cells membrane activity (Focal adhesion points), however longer treatment (day 7) with Dex impaired the migratory speed and impacting the homing of hMSCs [32]. However, the upregulated migration may be attributed due to higher expression of PGE-2 after Dex preconditioning. Lu et al. [38] study indicates that PGE-2 facilitates hMSCs migration and their findings suggest that EP2 prostanoid receptor encourages hMSCs migration through activation of FAK and ERK1/2 pathways. These data suggest that Dex may have various effects on the actin dynamics of hMSCs, with possible effects on its migratory activity [32, 38].

Taken together, robust stemness and enhanced migratory property indicates the higher regenerative potential, better survival in vivo, maintaining the primitive stage of hMSCs, and homing of tissue specific hMSCs. Since immunoregulatory mechanisms can vary between different species and hMSCs tissue sources, a variety of factors could participate in the hMSCs immunosuppression mechanisms. Reported findings suggest that IL-10 and TGF-β1 may not have a major role in BM-MSC immunoregulation [39]. However, both cell–cell contact and paracrine signaling mechanisms are implied in the immunoregulatory functions of hMSCs. Paracrine signaling and immunomodulation mechanisms are mainly governed by various molecules such as IDO, iNOS, IL-6, and COX-2, and HLA-G1/G5 eliciting different mechanisms on immune cells [39]. Therefore, we again for the first time have evaluated the time-dependent, tissue specific hMSCs response to different concentrations of Dex, evaluated by immunomodulatory genes PGE-2, IDO and HLA-G. Prostaglandins are small molecule derivatives of arachidonic acid (AA), has a property to furnish toward immune pathology and creates a potential target for immunomodulation. Notably, the effect of PGE-2 in most cases is exerted in combination with other immunosuppressive molecules [38]. Alongside its role in inflammatory response, PGE-2 is also involved in proliferation and migration in several cell types. Therefore, we looked for the gene expression level of PGE-2 in BM-MSCs, AD-MSCs, DP-MSCs, and UC-MSCs after pre-treatment with Dex (1000, 2000, 3000 ng/ml) at different time points. Interestingly, PGE-2 expression was significantly high in BM-MSCs and DP-MSCs at 1000 ng/ml at 24 h. Whereas, AD-MSCs and UC-MSCs did not show any significant response to Dex concentrations and time points. Tryptophan depleting enzyme indo-leamine-2,3-dioxygenase (IDO) is considered as one of the major molecules for mediating hMSCs immune suppression. Distinctively, IDO is the first and rate-limiting enzyme involved in degradation of tryptophan down the kynurenine pathway and is mostly expressed in antigen presenting cells (APCs) in response to IFN-gamma [40]. However, IDO is also majorly expressed in the placental cells and is responsible for fetal-maternal tolerance [41]. Herein, we sought for the effect of Dex preconditioning upon IDO expression in hMSCs at gene and protein level. It was observed that at gene level BM-MSCs and AD-MSCs showed the significant upregulation of IDO at 1000 ng/ml for 24 h. Whereas, assessment of IDO activity in conditioned media for BM-MSCs showed dose and time dependent upregulation, i.e., 3000 ng/ml for 48 h showed the highest IDO activity. Moreover, to the best of our knowledge this is the novel study, where we showed the upregulation of IDO after Dex preconditioning alone. However, other groups have used glucocorticoid steroids such as budesonide for augmenting IDO expression and activity in hMSCs [42].

Prior to this study, there have been only few reports about the effect of glucocorticoids (GCs) on the expression of HLA-G, and to the best of our knowledge, there are no reports on the association of Dex effect on hMSCs and HLA-G. Therefore, we evaluated the gene and protein HLA-G expression in tissue specific hMSCs and observed that at gene level there is no significant difference. In addition to this, the surface expression of HLA-G1, intracellular expression of HLA-G1/G5, and soluble HLA-G5 were investigated with all concentrations (1000, 2000, 3000 ng/ml) of Dex at 24 h. We observed that BM-MSCs showed significant HLA-G1, G1/G5, and G5 expression at 1000 ng/ml, AD, and DP-MSCs showed the highest expression of surface and intracellular HLA-G at 2000 ng/ml whereas soluble HLA-G is highly expressed at 3000 ng/ml. Among all the tissue specific hMSCs, UC-MSCs showed the lowest amount of upregulation of all forms of HLA-G. However, the variation in the response of tissue specific hMSCs is might be due to higher basal expression level of HLA-G in maternal associated tissue than the other tissue specific hMSCs. This indicates that UC-MSCs showed minimal response to Dex whereas BM-MSCs, AD-MSCs, and DP-MSCs showed the highest expression level in a dose-dependent manner. Moreau et al. showed that Dex and hydrocortisone up-regulate HLA-G in first-trimester trophoblast cells at the gene level [43–45]. Altogether we found that tissue specific hMSCs have differential response towards the dose and time point of Dex. Further we performed the functionality assessment (MLR) to understand the response of Dex preconditioned hMSCs. Interestingly, Michelo et al. reported that Dex did not hamper the immunosuppressive ability of hMSCs, on the other hand, it augmented the inhibitory effect of hMSCs via STAT5 phosphorylation, CD69 surface expression, and IFN-γ production. This urges that hMSCs and Dex are using the corresponding mode of action for suppression [44, 46]. However, it was observed that UC-MSCs and DP-MSCs showed the maximum amount of suppression followed by AD-MSCs and BM-MSCs.

Taken together, Dex preconditioning in tissue specific hMSCs showed upregulation in stemness and migratory property. In addition, it augments the therapeutic capacity, evidently via enhanced functional characteristics as well as immunosuppressive ability. However, higher concentration and shorter treatment duration (3000 ng/ml/24 h), low concentration for a longer treatment duration (1000 ng/ml/48 h) approach was seen to be an ideal setting to augment or upregulate the immunomodulatory response of these tissue specific hMSCs. Clinical application of this approach can simply involve exposure of UC/DP-MSCs to Dex for a shorter duration, before cell infusion.

Furthermore, preconditioning of hMSCs could be an interesting approach to maximize the systemic immunomodulatory effects of hMSCs. Enhancing the potency of a single hMSCs warrants the fewer hMSCs to be administered to achieve the similar therapeutic effect and allows them to endeavor the significant impact on its microenvironment.

## Conclusions

Our results provide evidence that low dose preconditioning for the longer time period and high dose preconditioning for a shorter time period of tissue specific hMSCs with dexamethasone maintains the native hMSCs properties viz enhancing the stemness, migration and immunomodulatory property. However, it should be noted that each tissue specific hMSCs respond differentially to Dex and elicit immunomodulatory factors, possibly impacting the success of stem cell treatment. Interestingly, as compared to other wide range of corticosteroids the Dex preconditioning did not significantly impacted the viability, metabolic activity, and morphology of hMSCs. Due to distinct mechanisms of action, the preconditioning of hMSCs with Dex may offer a promising therapeutic regimen for the enhancement of solid graft survival, potentially for the treatment of GvHD and now COVID-19. Therefore, our in vitro study demonstrated that tissue specific hMSCs responds in a dose-dependent manner and have no negative effect on hMSCs. Finally, for the purposes of a future clinical application, mechanistic pathways and the in vivo assessment are necessary to study the potential role of hMSCs for the treatment of various inflammatory disorders.

## Supplementary Information

**Additional file 1**.** Supplementary Figure 1**: A representative pictograph shows trilineage differentiation of tissue specific hMSCs (a) A panel shows osteocyte differentiation which is confirmed by alizarin red staining, (b) Adipocyte’s differentiation shows positivity for oil red “o” staining. (c) Chondrocyte’s differentiation shows positivity for Alcian blue staining. Scale bar 50 μm.** Supplementary Figure 2**: A representative pictograph showing the morphology of tissue specific hMSCs upon pre-conditioning of different Dex concentrations at 24 h & 48 h. Scale bar 100 μm.** Supplementary Figure 3**: A representative pictograph shows scratch assay in tissue specific hMSCs (a-d) Scratch at 0 h represents the initial day whereas closure of the area was taken at 12 h and 24 h and % open area was calculated. Scale bar 100 μm.** Supplementary Figure 4**: (a–d) Representative images shows intracellular SOX-2 expression level in BM-MSCs, whereas (e) Bar graph represent the relative intensity for SOX-2 expression after pre-conditioning with different Dex concentrations at 48 h, where 2000 ng/ml shows the significant expression level. (f–i) Representative images shows intracellular SOX-2 expression level in AD-MSCs, (j) Bar graph represent the relative intensity for SOX-2 expression after pre-conditioning with different Dex concentrations at 48 h and shows dose-dependent increase in expression level, (k–n) Representative images shows intracellular SOX-2 expression level in DP-MSCs, whereas (o) Bar graph represent the relative intensity for SOX-2 expression after pre-conditioning with different Dex concentrations at 48 h, (p–s) Representative images shows intracellular SOX-2 expression level in UC-MSCs, whereas (e) Bar graph represent the relative intensity for SOX-2 expression after pre-conditioning with different Dex concentrations at 48 h, shows dose-dependent increase in expression level. Scale bar 200 μm.** Supplementary Figure 5**: (a–d) Representative images shows intracellular NANOG expression level in BM-MSCs,whereas (e) Bar graph represent the relative intensity for NANOG expression after pre-conditioning with different Dex concentrations at 48 h, where 1000 ng/ml shows the significant expression level. (f–i) Representative images shows intracellular NANOG expression level in AD-MSCs, (j) Bar graph represent the relative intensity for NANOG expression after pre-conditioning with different Dex concentrations at 48 h and where 1000 ng/ml shows the significant expression level, (k–n) Representative images shows intracellular NANOG expression level in DP-MSCs, whereas (o) Bar graph represent the relative intensity for NANOG expression after pre-conditioning with different Dex concentrations at 48 h, where 3000 ng/ml shows the significant expression level (p–s) Representative images shows intracellular NANOG expression level in UC-MSCs, whereas (e) Bar graph represent the relative intensity for NANOG expression after pre-conditioning with different Dex concentrations at 48 h, shows dose-dependent increase in expression level. Scale bar 200 μm.** Supplementary Figure 6**: (a–d) Representative images shows intracellular OCT-4 expression level in BM-MSCs, whereas (e) Bar graph represent the relative intensity for OCT-4 expression after pre-conditioning with different Dex concentrations at 48 h, where 3000 ng/ml shows the significant expression level. (f–i) Representative images shows intracellular OCT-4 expression level in AD-MSCs, (j) Bar graph represent the relative intensity for OCT-4 expression after pre-conditioning with different Dex concentrations at 48 h and where 3000 ng/ml shows the significant expression level, (k–n) Representative images shows intracellular OCT-4 expression level in DP-MSCs, whereas (o) Bar graph represent the relative intensity for OCT-4 expression after pre-conditioning with different Dex concentrations at 48 h, where 2000 ng/ml shows the significant expression level (p–s) Representative images shows intracellular OCT-4 expression level in UC-MSCs, whereas (e) Bar graph represent the relative intensity for OCT-4 expression after pre-conditioning with different Dex concentrations at 48 h, where 1000 ng/ml shows the significant expression level. Scale bar 200 μm.

## Data Availability

Please contact the corresponding author for data requests.

## Abbreviations

Dex: Dexamethasone
hMSCs: Human mesenchymal stem cells
BM: Bone marrow
AD: Adipose tissue
DP: Dental pulp
UC: Umbilical cord
GvHD: Graft versus host disease
ISD: Immunosuppressive drugs
CsA: Cyclosporine
IMPDH: Mycophenolate mofetil dehydrogenase
COX: Cyclooxygenase
PGE-2: Prostaglandin-2
ESC: Embryonic stem cells
OD: Optical density
PBMCs: Peripheral blood mononuclear cells
PHA: Phytohemagglutinin A
GC: Glucocorticoids
