# Supplementary material for: Dexamethasone priming enhances stemness and immunomodulatory property of tissue-specific human mesenchymal stem cells
Source: BMC Dev Biol. 2021 Nov 4;21:16. doi: 10.1186/s12861-021-00246-4 (PMC8567134; doi:10.1186/s12861-021-00246-4)
Supplement: Supplementary file 1 — Additional file 1. Supplementary Figure 1: A representative pictograph shows trilineage differentiation of tissue specific hMSCs (a) A panel shows osteocyte differentiation which is confirmed by alizarin red staining, (b) Adipocyte’s differentiation shows positivity for oil red “o” staining. (c) Chondrocyte’s differentiation shows positivity for Alcian blue staining. Scale bar 50 μm. Supplementary Figure 2: A representative pictograph showing the morphology of tissue specific hMSCs upon pre-conditioning of different Dex concentrations at 24 h & 48 h. Scale bar 100 μm. Supplementary Figure 3: A representative pictograph shows scratch assay in tissue specific hMSCs (a-d) Scratch at 0 h represents the initial day whereas closure of the area was taken at 12 h and 24 h and % open area was calculated. Scale bar 100 μm. Supplementary Figure 4: (a–d) Representative images shows intracellular SOX-2 expression level in BM-MSCs, whereas (e) Bar graph represent the relative intensity for SOX-2 expression after pre-conditioning with different Dex concentrations at 48 h, where 2000 ng/ml shows the significant expression level. (f–i) Representative images shows intracellular SOX-2 expression level in AD-MSCs, (j) Bar graph represent the relative intensity for SOX-2 expression after pre-conditioning with different Dex concentrations at 48 h and shows dose-dependent increase in expression level, (k–n) Representative images shows intracellular SOX-2 expression level in DP-MSCs, whereas (o) Bar graph represent the relative intensity for SOX-2 expression after pre-conditioning with different Dex concentrations at 48 h, (p–s) Representative images shows intracellular SOX-2 expression level in UC-MSCs, whereas (e) Bar graph represent the relative intensity for SOX-2 expression after pre-conditioning with different Dex concentrations at 48 h, shows dose-dependent increase in expression level. Scale bar 200 μm. Supplementary Figure 5: (a–d) Representative images shows intracellular NANOG e [file 12861_2021_246_MOESM1_ESM.docx]

**Dexamethasone priming enhances stemness and immunomodulatory property of tissue-specific human mesenchymal stem cells**

**Sonali Rawat ^1^, Vatsla Dadhwal^2^, Sujata Mohanty ^1^***

^1^Stem Cell Facility, All India Institute of Medical Science; [sonali26rawat@gmail.com](mailto:sonali26rawat@gmail.com) (ORCID ID: https://orcid.org/0000-0001-6518-3547)

^2^Department of Obstetrics and Gynecology, All India Institute of Medical Science; vatslad@aiims.edu

***Correspondence: drmohantysujata@gmail.com; Tel.: (+91-9810291336)**

**
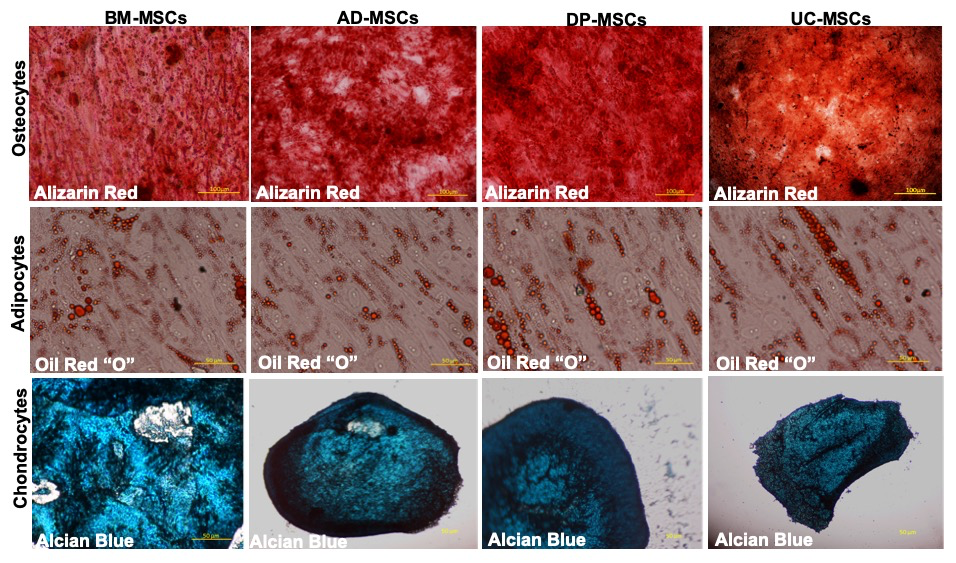
**

**Supplementary Figure 1:** A representative pictograph shows trilineage differentiation of tissue specific hMSCs (a) A panel shows osteocyte differentiation which is confirmed by alizarin red staining, (b) Adipocyte’s differentiation shows positivity for oil red “o” staining. (c) Chondrocyte’s differentiation shows positivity for Alcian blue staining. Scale bar 50µm.


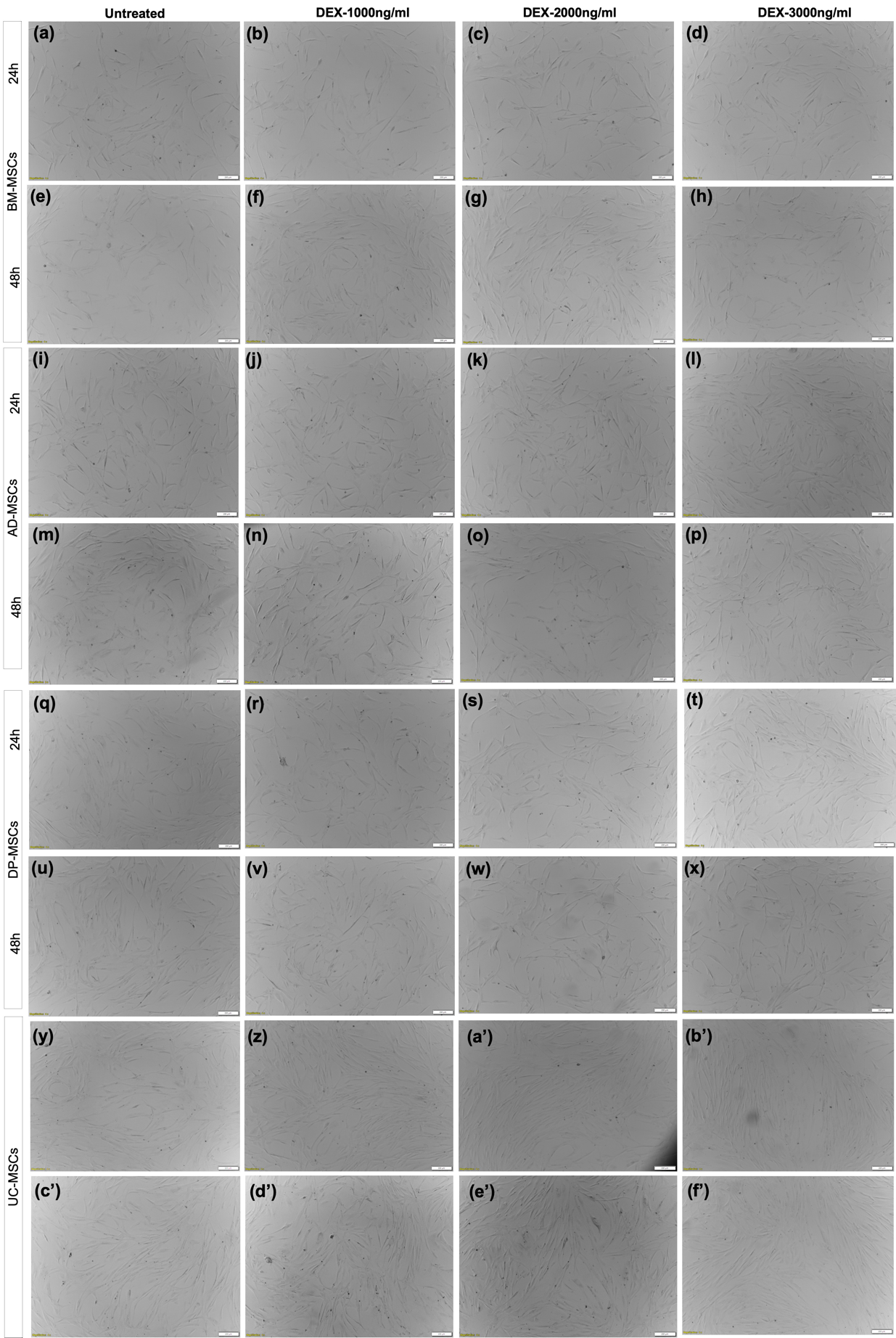


**Supplementary Figure 2:**  A representative pictograph showing the morphology of tissue specific hMSCs upon pre-conditioning of different Dex concentrations at 24 h & 48 h. Scale bar 100 µm


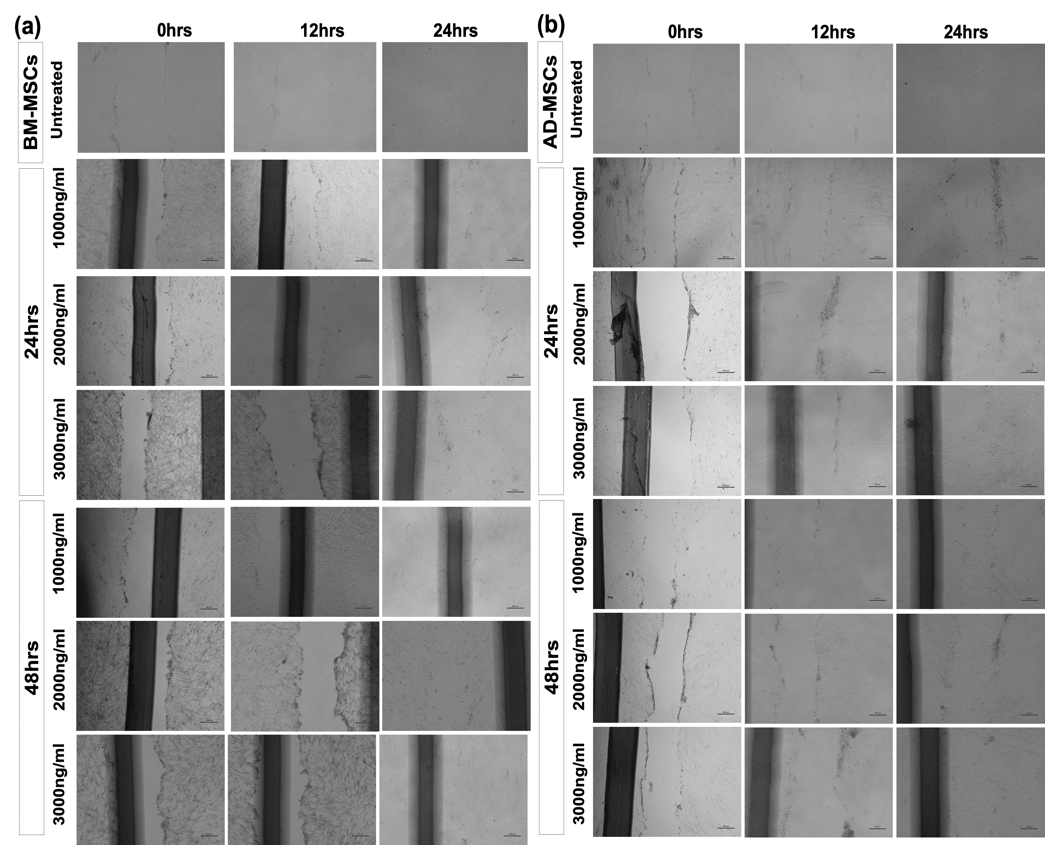


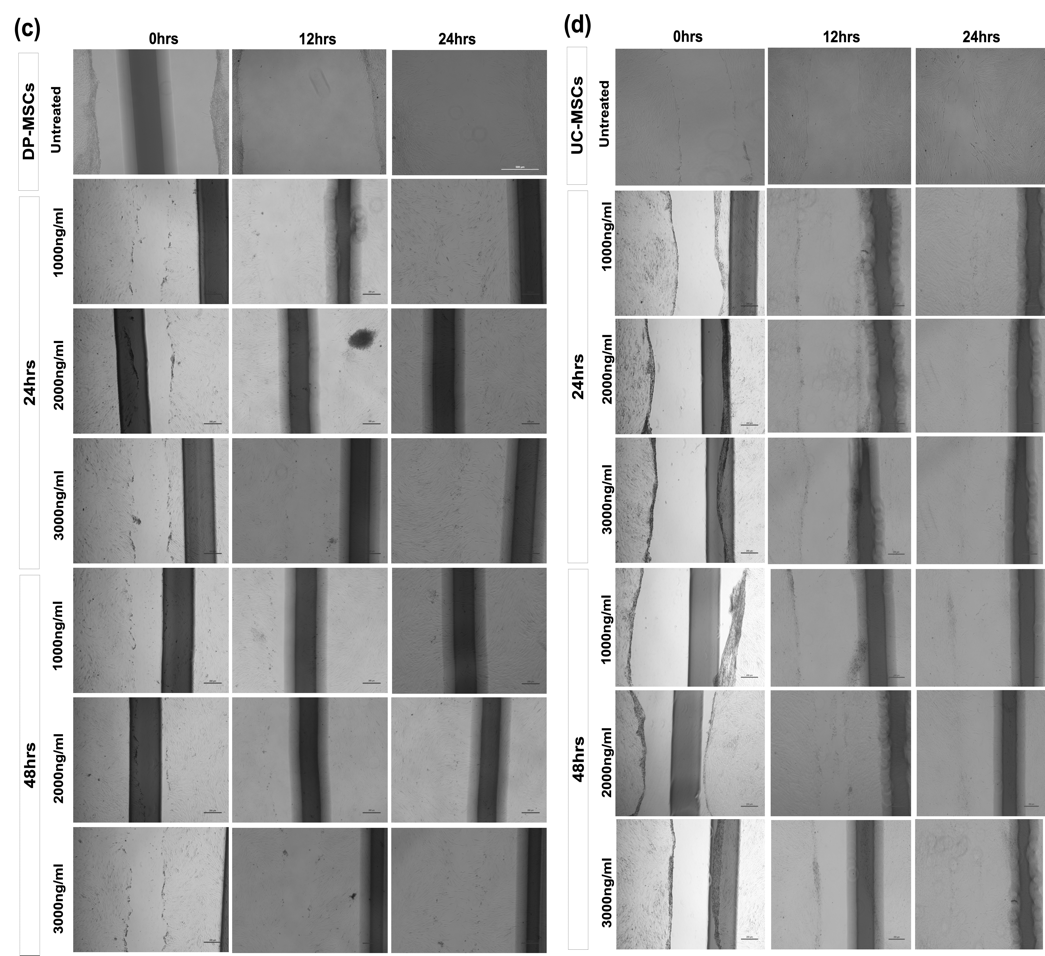


**Supplementary Figure 3:** A representative pictograph shows scratch assay in tissue specific hMSCs (a-d) Scratch at 0h represents the initial day whereas closure of the area was taken at 12 h and 24 h and % open area was calculated. Scale bar 100 µm.


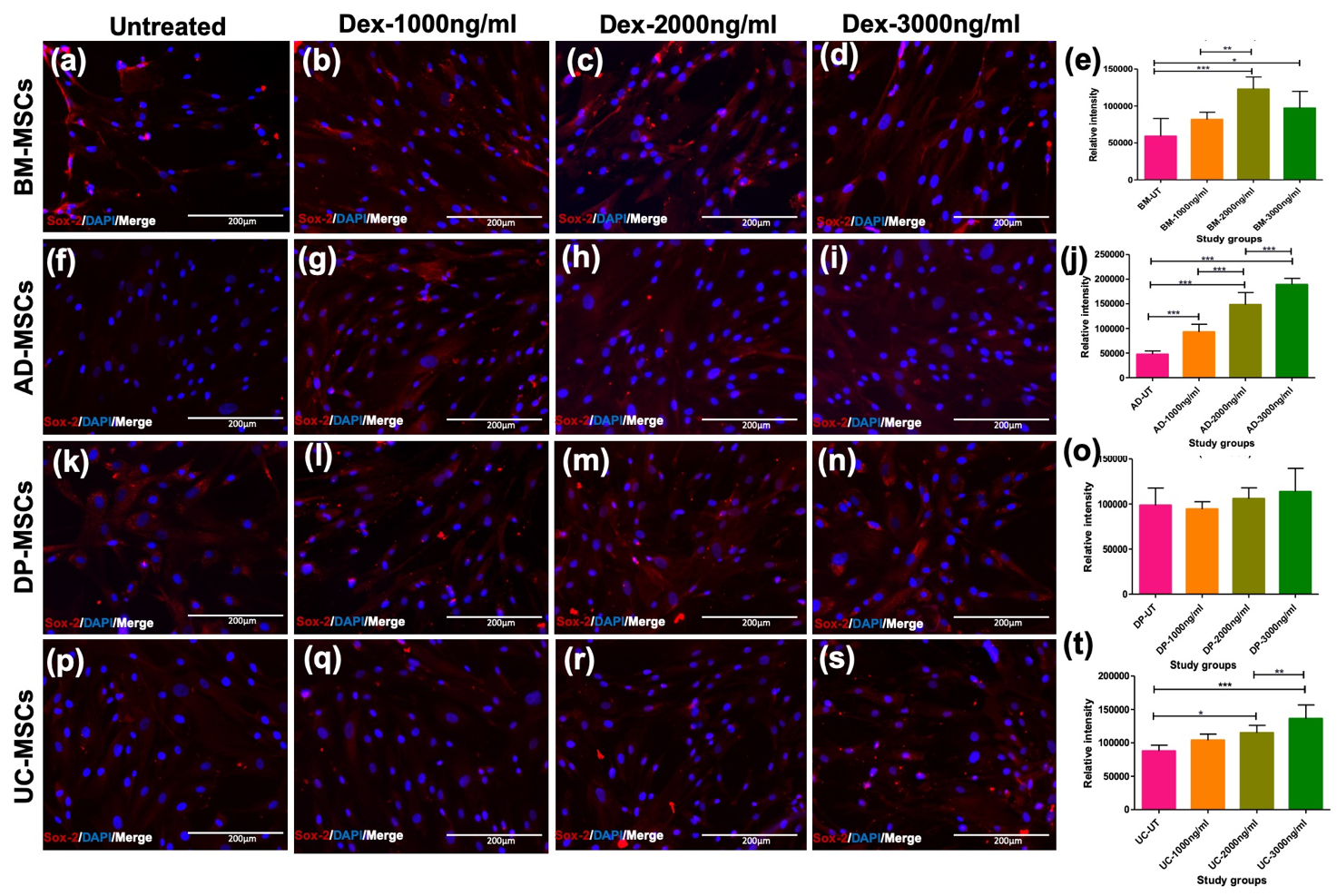


**Supplementary Figure 4:** (a-d) Representative images shows intracellular SOX-2 expression level in BM-MSCs, whereas (e) Bar graph represent the relative intensity for SOX-2 expression after pre-conditioning with different Dex concentrations at 48h, where 2000 ng/ml shows the significant expression level. (f-i) Representative images shows intracellular SOX-2 expression level in AD-MSCs, (j) Bar graph represent the relative intensity for SOX-2 expression after pre-conditioning with different Dex concentrations at 48h and shows dose-dependent increase in expression level, (k-n) Representative images shows intracellular SOX-2 expression level in DP-MSCs, whereas (o) Bar graph represent the relative intensity for SOX-2 expression after pre-conditioning with different Dex concentrations at 48h, (p-s) Representative images shows intracellular SOX-2 expression level in UC-MSCs, whereas (e) Bar graph represent the relative intensity for SOX-2 expression after pre-conditioning with different Dex concentrations at 48h, shows dose-dependent increase in expression level. Scale bar 200 µm.


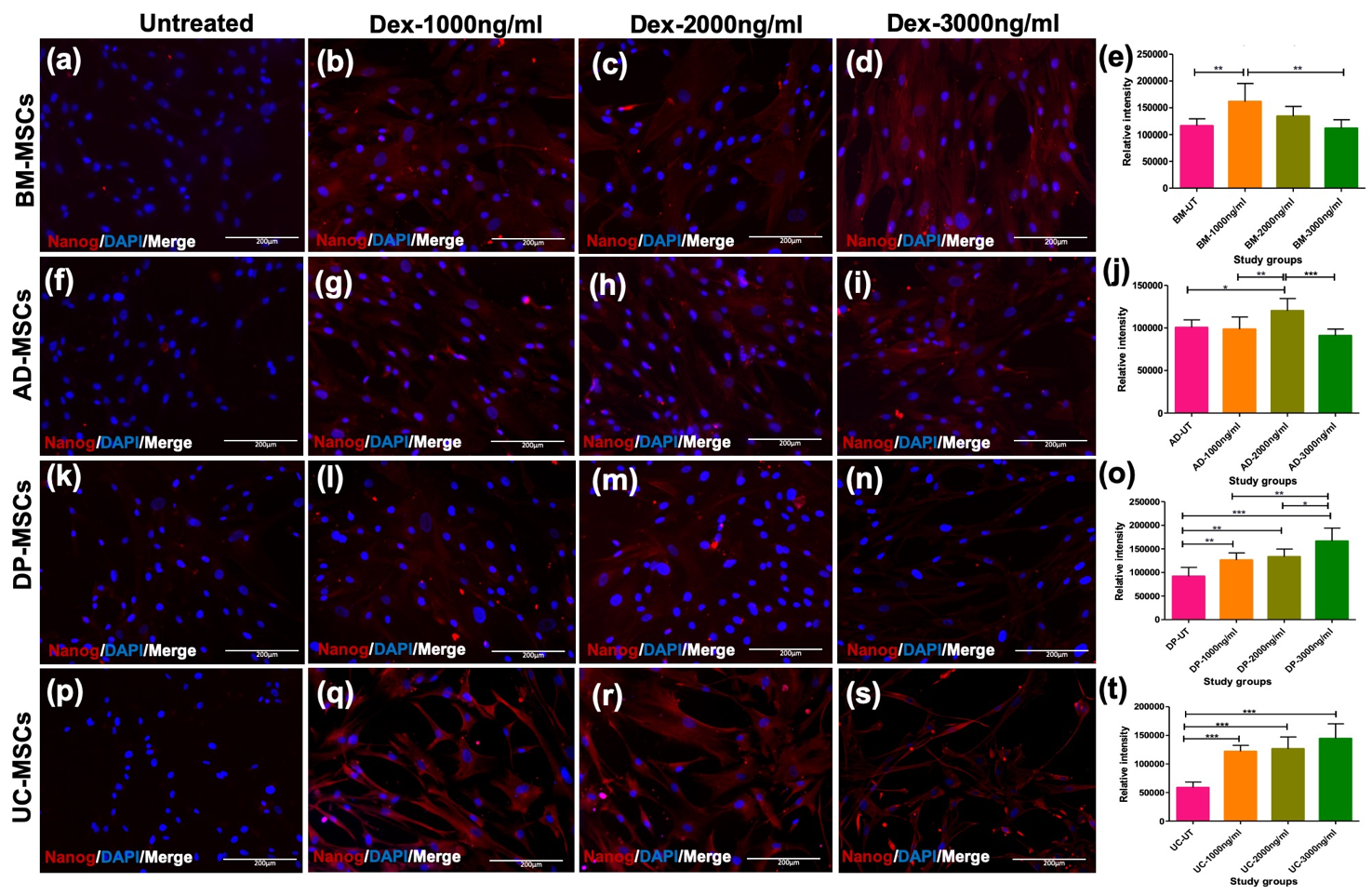


**Supplementary Figure 5:** (a-d) Representative images shows intracellular NANOG expression level in BM-MSCs, whereas (e) Bar graph represent the relative intensity for NANOG expression after pre-conditioning with different Dex concentrations at 48h, where 1000ng/ml shows the significant expression level. (f-i) Representative images shows intracellular NANOG expression level in AD-MSCs, (j) Bar graph represent the relative intensity for NANOG expression after pre-conditioning with different Dex concentrations at 48h and where 1000 ng/ml shows the significant expression level, (k-n) Representative images shows intracellular NANOG expression level in DP-MSCs, whereas (o) Bar graph represent the relative intensity for NANOG expression after pre-conditioning with different Dex concentrations at 48h, where 3000 ng/ml shows the significant expression level (p-s) Representative images shows intracellular NANOG expression level in UC-MSCs, whereas (e) Bar graph represent the relative intensity for NANOG expression after pre-conditioning with different Dex concentrations at 48h, shows dose-dependent increase in expression level. Scale bar 200 µm.


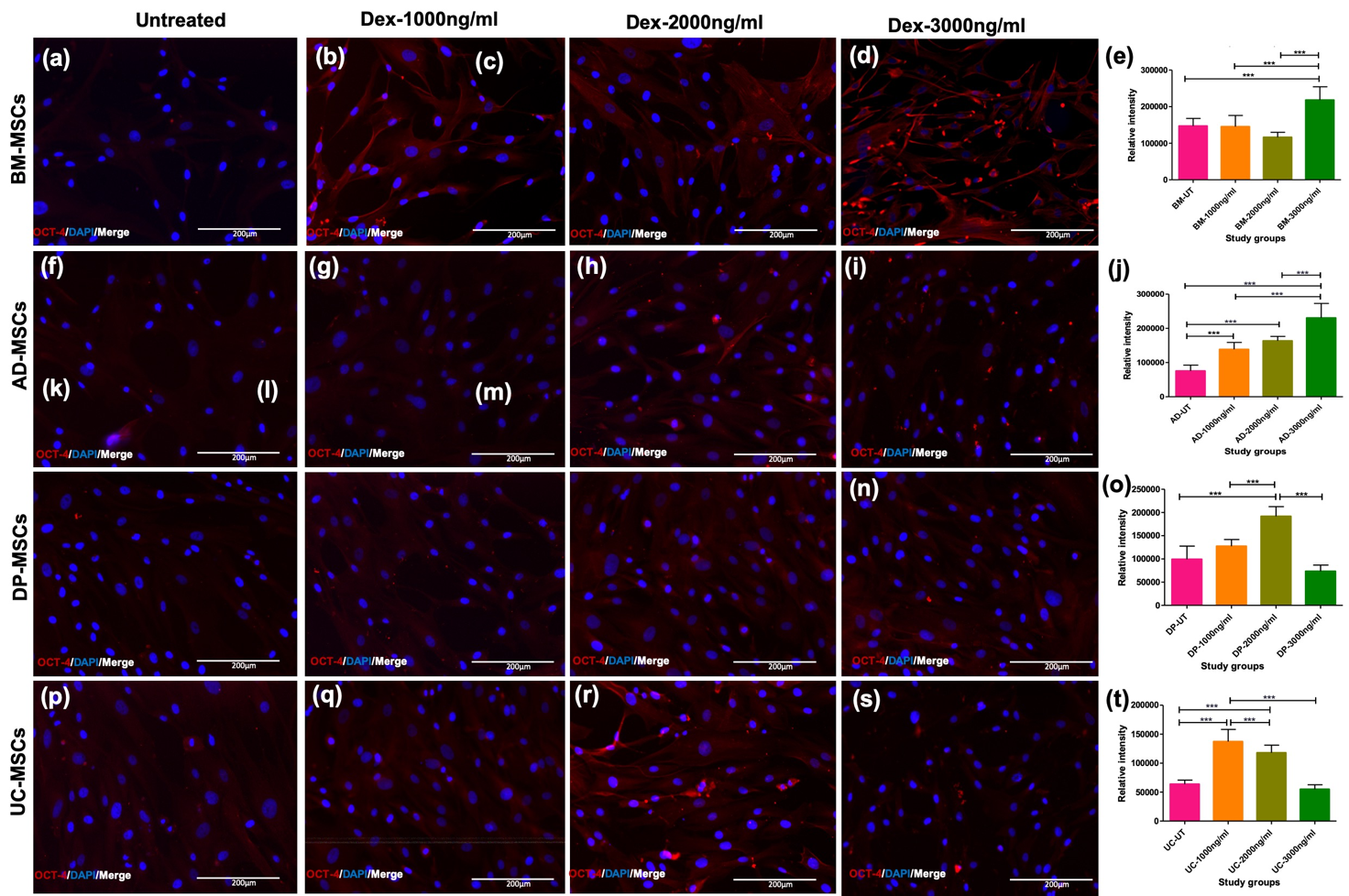


**Supplementary Figure 6:** (a-d) Representative images shows intracellular OCT-4 expression level in BM-MSCs, whereas (e) Bar graph represent the relative intensity for OCT-4 expression after pre-conditioning with different Dex concentrations at 48h, where 3000ng/ml shows the significant expression level. (f-i) Representative images shows intracellular OCT-4 expression level in AD-MSCs, (j) Bar graph represent the relative intensity for OCT-4 expression after pre-conditioning with different Dex concentrations at 48h and where 3000 ng/ml shows the significant expression level, (k-n) Representative images shows intracellular OCT-4 expression level in DP-MSCs, whereas (o) Bar graph represent the relative intensity for OCT-4 expression after pre-conditioning with different Dex concentrations at 48h, where 2000 ng/ml shows the significant expression level (p-s) Representative images shows intracellular OCT-4 expression level in UC-MSCs, whereas (e) Bar graph represent the relative intensity for OCT-4 expression after pre-conditioning with different Dex concentrations at 48h, where 1000ng/ml shows the significant expression level. Scale bar 200 µm.
